# SMC5/6 acts jointly with Fanconi anemia factors to support DNA repair and genome stability

**DOI:** 10.15252/embr.201948222

**Published:** 2019-12-23

**Authors:** Francesco Rossi, Anne Helbling‐Leclerc, Ryotaro Kawasumi, Nanda Kumar Jegadesan, Xinlin Xu, Pierre Devulder, Takuya Abe, Minoru Takata, Dongyi Xu, Filippo Rosselli, Dana Branzei

**Affiliations:** ^1^ The FIRC Institute of Molecular Oncology IFOM Milan Italy; ^2^ UMR8200 CNRS Equipe Labellisée La Ligue Contre le Cancer Université Paris Sud Gustave Roussy Villejuif Cedex France; ^3^ School of Life Sciences Peking University Beijing China; ^4^ Laboratory of DNA Damage Signaling Radiation Biology Center Graduate School of Biostudies Kyoto University Kyoto Japan; ^5^ Istituto di Genetica Molecolare Consiglio Nazionale delle Ricerche (IGM‐CNR) Pavia Italy; ^6^ Present address: Department of Chemistry Graduate School of Science Tokyo Metropolitan University Hachioji‐shi Tokyo Japan

**Keywords:** DNA repair, Fanconi anemia, intra‐ and inter‐strand crosslinks, mitotic instability, SMC5/6, Cell Cycle, DNA Replication, Repair & Recombination, Molecular Biology of Disease

## Abstract

SMC5/6 function in genome integrity remains elusive. Here, we show that SMC5 dysfunction in avian DT40 B cells causes mitotic delay and hypersensitivity toward DNA intra‐ and inter‐strand crosslinkers (ICLs), with *smc5* mutants being epistatic to FANCC and FANCM mutations affecting the Fanconi anemia (FA) pathway. Mutations in the checkpoint clamp loader RAD17 and the DNA helicase DDX11, acting in an FA‐like pathway, do not aggravate the damage sensitivity caused by SMC5 dysfunction in DT40 cells. SMC5/6 knockdown in HeLa cells causes MMC sensitivity, increases nuclear bridges, micronuclei, and mitotic catastrophes in a manner similar and non‐additive to FANCD2 knockdown. In both DT40 and HeLa systems, SMC5/6 deficiency does not affect FANCD2 ubiquitylation and, unlike FANCD2 depletion, RAD51 focus formation. SMC5/6 components further physically interact with FANCD2‐I in human cells. Altogether, our data suggest that SMC5/6 functions jointly with the FA pathway to support genome integrity and DNA repair and may be implicated in FA or FA‐related human disorders.

## Graphical abstract

The structural maintenance complex SMC5/6 interacts with and functions jointly with key components of the Fanconi anemia pathway to facilitate DNA repair and preserve genome stability.

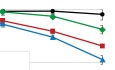

## Introduction

Genomic integrity is safeguarded by multiple genome caretakers that are often implicated in chromosome metabolism reactions induced by various types of replication stress. The structural maintenance of chromosomes (SMC) complexes, including cohesin (SMC1/3), condensin (SMC2/4), and SMC5/6, are critical for chromosome transactions that ensure normal proliferation and genome integrity. Structurally, SMC complexes form molecular rings that can entrap genomic DNA 1. The SMC5/6 complex ring is composed of two coiled‐coil SMC heterodimers SMC5 and SMC6, which associate with a kleisin component, NSMCE4 2. In addition, SMC5/6 contains several peripheral subunits. The NSMCE1–NSMCE3 heterodimer has ubiquitin ligase activity and interacts with NSMCE4 and SMC6, while NSMCE2 has SUMO ligase activity and interacts with SMC5 3. The NSMCE1‐NSMCE3‐NSMCE4 sub‐complex presents double‐stranded (ds) DNA‐binding activity, without preference for structured DNA 4. SMC5/6 also interacts with SLF1 and SLF2, which are functional orthologs of budding and fission yeast Nse5 and Nse6, respectively 5. The budding yeast Nse5/6 heterodimer is a component of the Smc5/6 complex (essential for proliferation in budding yeast, but not in fission yeast) and has single‐stranded (ss) DNA‐binding activity 6. In human cells, SLF1 and SLF2 physically link RAD18 to the SMC5/6 complex, defining a pathway for SMC5/6 recruitment to sites of DNA damage 5.

In terms of molecular functions, SMC5/6 contributes to DNA repair 7, facilitates DNA topological transitions 8, and promotes timely resolution of DNA cruciform structures arising during homologous recombination repair 9, 10. In unperturbed conditions, budding yeast Smc5/6 is required for the organization and segregation of repeat elements 1, 11, 12 and promotes replication through difficult to replicate regions known as natural pausing sites 13.

Understanding the functions of SMC5/6 in genome integrity and DNA repair is important, because mutations in SMC5/6 have been linked to different human disease conditions that may derive from impaired DNA metabolism reactions. Specifically, mutations in SMC5/6 components increase overall breast cancer risk 14, promote brain metastasis development 15, and result in debilitating diseases associated with severe developmental defects. To date, two genetic disorders caused by mutations in SMC5/6 have been reported: the NSMCE2‐associated syndrome featuring primordial dwarfism and deregulation of glucose metabolism 16, and the NSMCE3‐associated disorder also known as LICS, characterized by increased chromosome breakage and defective T‐ and B‐cell function 17. At the cellular level, the two syndromes are characterized by increased replication stress, micronuclei formation, and defects in homologous recombination (HR). While the involvement of SMC5/6 in preventing replication‐stress accumulation is consistent with the results obtained in other model systems and likely linked to its function in DNA repair, the molecular roles of SMC5/6 in DNA repair are incompletely understood.

Here, we set out to investigate the roles of SMC5/6 that assist genome integrity in vertebrate and mammalian cells, by studying the consequences of SMC5/6 depletion in avian DT40 cells and HeLa cells. Both knockout and conditional depletion of SMC5 in DT40 cells allowed cellular proliferation, as previously reported 18. *smc5* mutant cells showed sensitivity toward DNA intra‐ and inter‐strand crosslinkers (ICLs). Notably, the repair defect of *smc5* DT40 cells toward cisplatin was genetically epistatic with mutations in the Fanconi anemia (FA) components, FANCC and FANCM, and with mutations in the FA‐like pathway defined by the DNA damage checkpoint clamp loader, RAD17, and the DDX11 helicase 19, 20. Moreover, *smc5* mutants were additive to mutations in *KU70*, mediating non‐homologous end‐joining (NHEJ) repair of double‐strand breaks (DSBs). SMC5/6 knockdown in human cells also caused ICL sensitivity, micronuclei, and aberrant mitoses, in a manner similar and non‐additive with FANCD2 knockdown 21. However, SMC5/6 dysfunction did not interfere with FANCD2 ubiquitylation, and unlike FANCD2 depletion, it did not affect RAD51 focus formation in unperturbed or replication‐stress conditions. Combining co‐immunoprecipitation and mass‐spectrometry approaches, we detected physical interaction between several SMC5/6 components and FANCD2‐I in human cells, both when SMC5/6 was overexpressed and with endogenous proteins. The results support the notion that vertebrate SMC5/6 functions jointly with the FA pathway in the repair of DNA lesions to prevent genome instability. We propose that mutations in SMC5/6 in humans may be implicated in FA or FA‐like disorders.

## Results

### SMC5/6 function is required for normal proliferation in DT40 cells

**Figure EV1:**
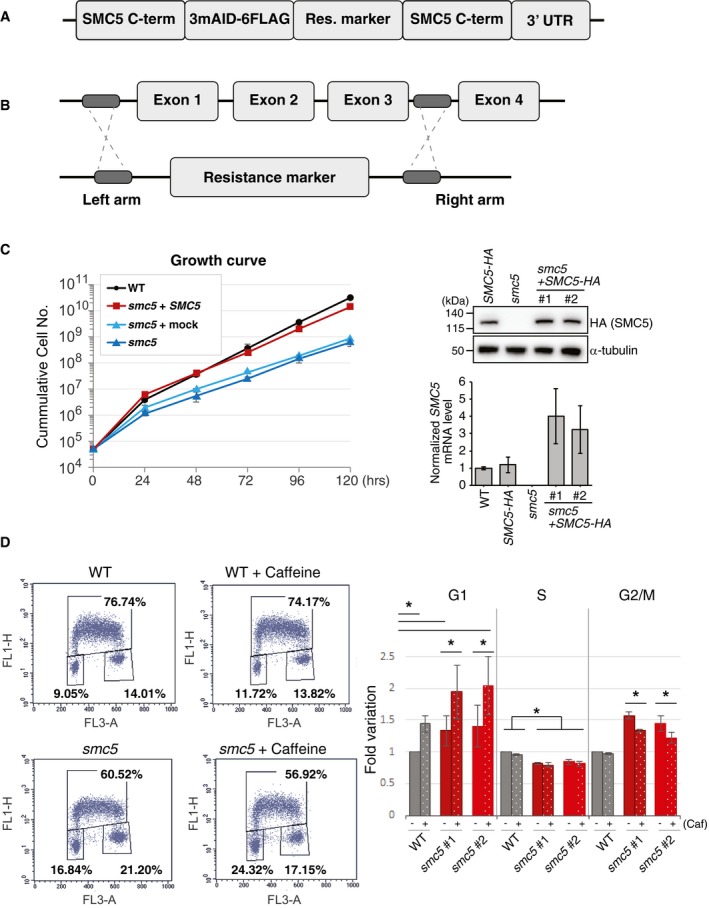
SMC5 function promotes normal cell cycle and prevents checkpoint‐dependent G2/M delay Schematic representation of the *smc5‐aid‐flag* modified genomic locus.Schematic representation of the *smc5* KO construct.Growth curve of WT and *smc5* cells complemented or not with chicken SMC5‐HA cDNA. The experiment is carried out at the indicated temperature of 39.5°C. The data represent the means ± SD of three experiments. On the right panel, WB analysis of SMC5‐HA expression after complementation. Alpha‐Tubulin is used as loading control.Bidimensional FACS analysis is carried out to study cell cycle distribution after caffeine treatment (+Caf). Data represent means ± SD of four experiments. Asterisks indicate *P* value ≤ 0.05, as calculated by paired *t*‐test.

**Figure 1:**
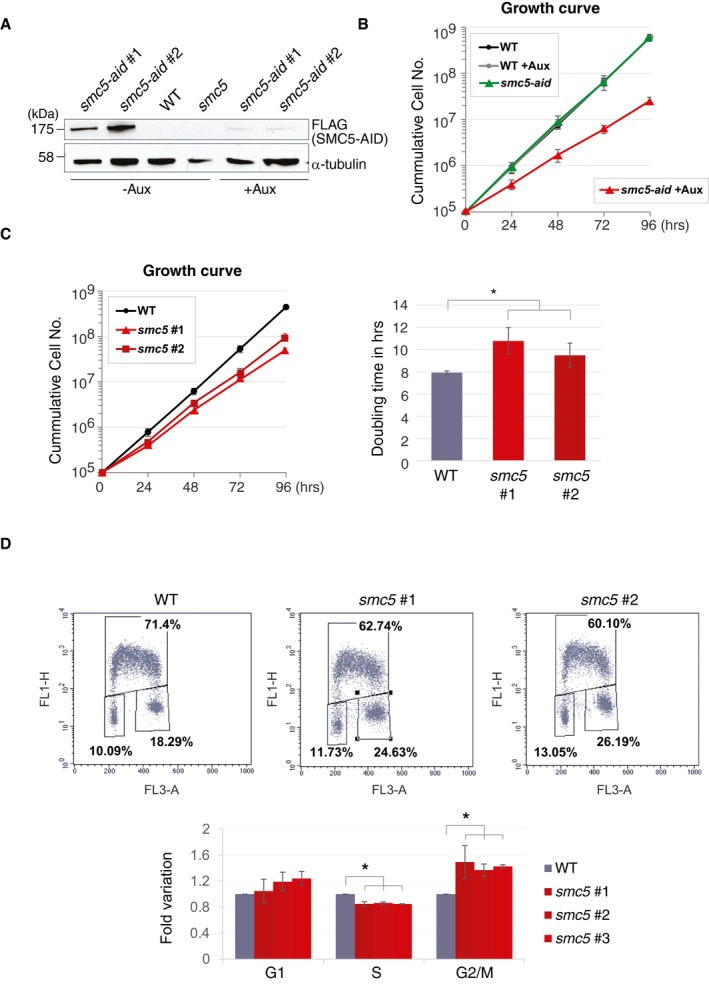
SMC5 dysfunction causes slower proliferation WB analysis of *smc5‐aid* clones in the absence or presence of auxin using α‐FLAG. Tubulin is used as loading control and WT cells are used as a negative control for the FLAG tag.Proliferation curve of WT and *smc5‐aid* cells in the presence or absence of auxin. The data represent means ± SD of three independent experiments.Proliferation curves of WT and two independent *smc5* clones grown at 39.5°C, with the estimated doubling time on the right. Data represent means ± SD of four experiments. Asterisks indicate *P* value ≤ 0.05, as derived from unpaired *t*‐test type 3, two‐sample unequal variance, carried out to check statistical significance between *smc5* clones and WT. *smc5* clone 1 vs. WT, *P* = 0.016, *smc5* clone 2 vs. WT, *P* = 0.04.Bidimensional FACS analysis shows significant accumulation in G2/M and decrease in the S phase in *smc5* mutants. The data represent means ± SD of three independent experiments for *smc5* clones 2 and 3, and 4 independent experiments for WT and *smc5* clone 1. Asterisks indicate *P* value ≤ 0.05, detailed below, using paired *t*‐test. S phase (*smc5* clones 1, 2, 3 vs. WT: *P* = 0.0031; *P* = 0.0002; *P* = 0.004, respectively). G2/M (*smc5* clones 1, 2, 3 vs. WT: *P* = 0.029; *P* = 0.0014; *P* = 0.02, respectively). Source data are available online for this figure.

Here, we applied the auxin‐inducible degron (AID) system, which enables rapid degradation of target proteins by the proteasome 22, 23 to establish conditional depletion of SMC5. We used the DT40 avian B‐cell line that stably expresses *TIR1*, an essential component in the auxin degron system 24 in which we C‐terminally tagged the endogenous *SMC5* gene with the 3AID‐6FLAG tag, using the Flip‐In system for insertion of epitope tags 25 (Fig EV1A). As *SMC5* is present on chromosome Z, with only one replacement we generated *SMC5* ^/*3AID6FLAG*^ cells expressing *TIR1* (hereafter referred to as *smc5‐aid*) cells. The functionality of the AID tag inserted to the carboxyl‐terminus of SMC5 was confirmed by Western blotting (Fig 1A). After auxin addition, the SMC5‐3AID‐6FLAG protein was strongly reduced within 24 h (Fig 1A). In regard to proliferation, *smc5‐aid* cells behaved similarly with the wild‐type (WT) control in the absence of auxin, but addition of auxin caused slower proliferation (Fig 1B).

To rule out that remaining low levels of SMC5‐AID are sufficient for proliferation, albeit with lower speed, we established constitutive knockout of *SMC5*, employing a construct that causes complete disruption of the open‐reading frame (Fig EV1B). *SMC5* knockout cells (hereafter referred to as *smc5*) proliferated slower than WT cells and had a longer doubling time (Fig 1C), showing results similar to the ones observed upon SMC5‐AID depletion.

We further investigated whether the observed slower proliferation phenotype of *smc5* cells is complemented by expressing WT copies of chicken SMC5 (c*SMC5*) tagged with HA. To estimate the level of introduced cSMC5‐HA vs. endogenous SMC5 present in WT, we also established an *smc5‐HA* cell line using the Flip‐In system and used both Western blotting against HA and quantification of mRNA levels (Fig EV1C). The results revealed that cSMC5‐HA introduced for complementation was expressed 3–4 times higher than endogenous SMC5 and fully complemented the growth defect of *smc5* cells (Fig EV1C). Next, we assessed the cell cycle distribution profile, and found a statistically significant increase in the G2/M population in *smc5* mutants (about 25% in *smc5* compared with 18% in WT), which was associated with a concomitant decrease in the S‐phase population (Fig 1D). We considered the scenario in which *smc5* mutants endure replication stress and as a result accumulate lesions that cause G2/M arrest and/or problems in completing mitosis. Supportive of this notion, addition of caffeine, which inhibits damage checkpoint kinases, reduced the population of *smc5* cells arrested in G2/M (Fig EV1D). Thus, *SMC5* is dispensable for viability in avian DT40 cells 18, but is required for physiological levels of fast proliferation.

### SMC5 acts jointly with the FA protein FANCC in the repair of ICLs

SMC5 function has been linked to HR repair in several model systems, including budding and fission yeast, proliferating germ cells of *Caenorhabditis elegans* and avian DT40 cells 9, 10, 18, 26, 27, 28, 29. In chicken DT40 cells, *smc5* mutants are sensitive to IR (ionizing radiation) and MMS (methyl methanesulfonate) 18. In regard to IR sensitivity, *smc5* cells are epistatic to *rad54*, defective in HR, but additive to *ku70*, defective in the non‐homologous end‐joining (NHEJ) repair of double‐strand breaks (DSBs) 18. However, more information on the roles of SMC5/6 in DNA repair in vertebrate cells is currently missing, especially in regard to lesions other than DSBs.

**Figure 2:**
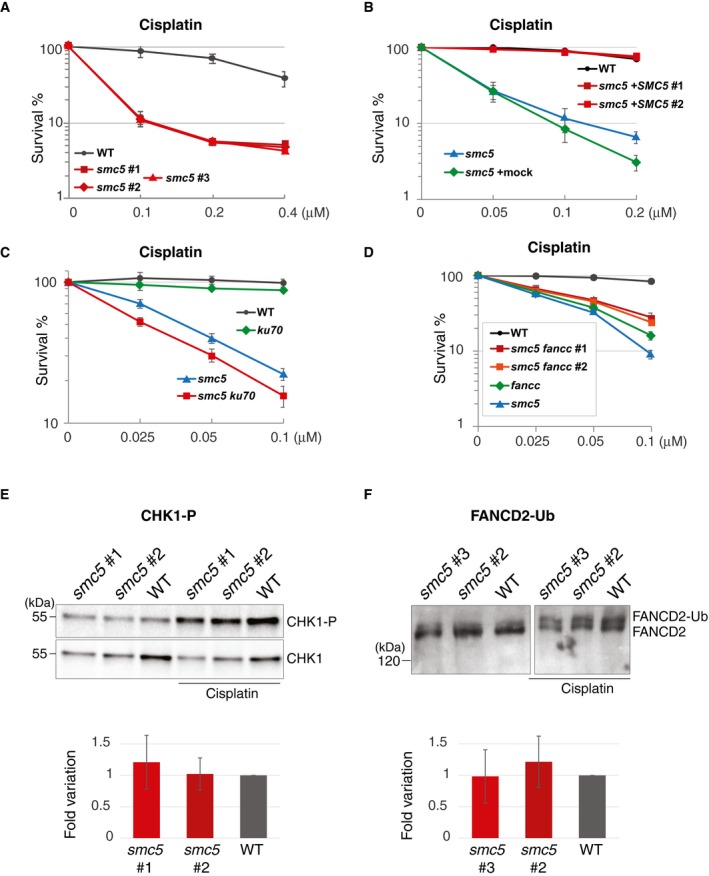
SMC5 contributes to the repair of intra‐ and inter‐strand crosslinks, independently of KU70 and jointly with FANCC Survival curve of *smc5* cells treated with different concentrations of cisplatin. Data represent means ± SD of three experiments.Survival curve of *smc5* cells complemented or not with chicken *SMC5* cDNA and treated with different concentrations of cisplatin. The experiment is carried out at 39.5°C. Data represent means ± SD of three experiments.Survival curve of *smc5 ku70* cells treated with different concentrations of cisplatin. Data represent means ± SD of three experiments.Survival curve of *smc5 fancc* cells treated with different concentrations of cisplatin. Data represent means ± SD of three experiments.WB analysis of CHK1‐P levels at steady state or after treatment with 1 μM cisplatin for 3 h. CHK1 total protein is used as loading control. Quantitative analysis of CHK1‐P levels at steady state vs. total CHK1 is shown in the bottom panel. Data represent means ± SD of four experiments.WB analysis of FANCD2 ubiquitylation levels at steady state after treatment with 1 μM cisplatin for 3 h. Analysis of the ratio between FANCD2 ubiquitylated/non‐ubiquitylated forms is shown on the bottom panel. Data represent means ± SD of three experiments. Source data are available online for this figure.

**Figure EV2:**
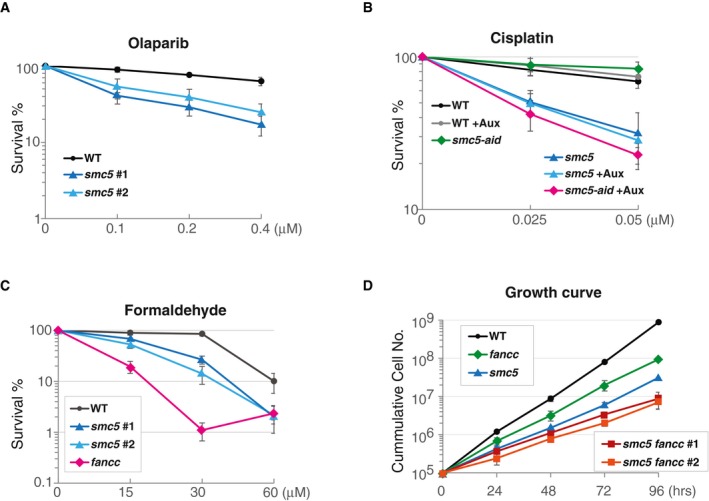
SMC5 promotes tolerance to olaparib, cisplatin, and formaldehyde A–CSurvival curve of cells of the indicated genotype toward various drugs. Data represent means ± SD of three experiments.DGrowth curve of cells of the indicated genotype. The data represent the means ± SD of three experiments.

We investigated the sensitivity of DT40 *smc5* mutants to several DNA damaging agents that cause replication‐associated lesions, using Celltiter‐Glo assay that measures the amount of ATP levels in living cells. We uncovered hypersensitivity of *smc5* cells toward cisplatin, a DNA damaging agent causing ICLs (Fig 2A). In addition, we observed intermediate levels of sensitivity of *smc5* cells toward the PARP inhibitor olaparib (Fig EV2A), which creates substrates relying on HR‐mediated functions for fork protection and/or fork restart 30, 31. The observed cisplatin sensitivity of *smc5* knockout cells was fully recovered by the expression of the *cSMC5‐HA* (Fig 2B, see also Fig EV1C for levels of expressed *cSMC5‐HA*) and also recapitulated in *smc5‐aid* cells treated with auxin (Fig EV2B). Similarly to cisplatin, endogenous formaldehyde causes ICLs 32. Thus, we next examined the sensitivity of *smc5* cells to this chemical. We found *smc5* mutants to be sensitive to formaldehyde (Fig EV2C), although to a lesser extent than *fancc* mutants defective in the FA pathway.

Previous reports found *smc5* mutants to be additive to mutations in *ku70*, defective in NHEJ, in regard to repair of lesions induced by IR 18. We found additivity between *smc5* and *ku70* knockout mutations also in regard to cisplatin (Fig 2C), while eliminating NHEJ suppresses the sensitivity of FA mutants 33, 34. Thus, SMC5 facilitates damage tolerance of bulky lesions and ICLs, and may be an important player, along with the FA pathway in mediating ICL repair.

Fanconi anemia is a multigenic syndrome characterized by bone marrow failure, developmental abnormalities, and predisposition to cancer, with FA patient cells showing a characteristic cellular sensitivity to agents that induce ICLs. So far, 22 complementation groups have been delineated in FA, with the causal genes shown to act in a common repair pathway 35, 36, 37. In addition, several FA components have also FA‐independent roles in DNA replication, fork protection, and checkpoint activation 38, 39, 40, 41.

The central components of the FA pathway, FANCD2 and FANCI, interact with each other 42, and are monoubiquitylated by the FANCL ubiquitin ligase component of the FA core complex, comprising several FA subunits (FA‐A, FA‐B, FA‐C, FA‐E, FA‐F, FA‐G, FA‐L, FA‐M, FA‐T) and associated proteins FAAP20 and FAAP100. FANCD2–FANCI ubiquitylation promotes lesion unhooking, causing the formation of a gapped DNA molecule, with the unhooked lesion in the gapped part, and a double‐strand break (DSB) on the other. The gap and DSB are subsequently repaired by TLS and HR 43. We investigated potential genetic relationships between SMC5 and FANCC, which is essential for FANCD2‐I ubiquitylation and required for both HR and TLS‐mediated pathways of DNA repair in DT40 cells 44. Both *fancc* and *smc5* mutants show mild proliferation delays with the double‐mutant *fancc smc5* showing additive growth defects (Fig EV2D). Double‐mutant *smc5 fancc* did not show additive sensitivity (Fig 2D), suggesting that SMC5 and FANCC do not work in redundant pathways. The apparent improved survival of the double mutant when exposed to cisplatin might stem from the poorer proliferation of these cells (Fig EV2D), which may cause them not to be effectively damaged by cisplatin that functions during replication.

Because FANCC is required for FANCD2 ubiquitylation, which also relies on an intact ATR checkpoint response 42, 45, we next analyzed whether these signaling pathways were functional in *smc5* mutants. We found that both CHK1 phosphorylation, used as readout for ATR activity, and FANCD2 ubiquitylation, used as readout of FA pathway functionality, proceeded unaffected in *smc5* mutants (Fig 2E and F). Thus, SMC5 functions downstream of FANCD2 ubiquitylation in a manner non‐redundant with FANCC, potentially to support the DNA repair function of the FA pathway.

### SMC5 acts jointly with FANCM and the FA‐like pathway involving the RAD17 checkpoint clamp loader and the DDX11 DNA helicase

FANCM is a component of the FA core presenting DNA translocase and branch‐migrating activities and a degenerate C‐terminal ERCC4 nuclease domain that binds to DNA structures *in vitro* 46. FANCM has functions within the FA pathway related to the recruitment of the FA core complex to chromatin and in triggering checkpoint activation, as well as functions outside the FA pathway related to lesion bypass potentially by fork reversal (reviewed in Ref. 47).

**Figure 3:**
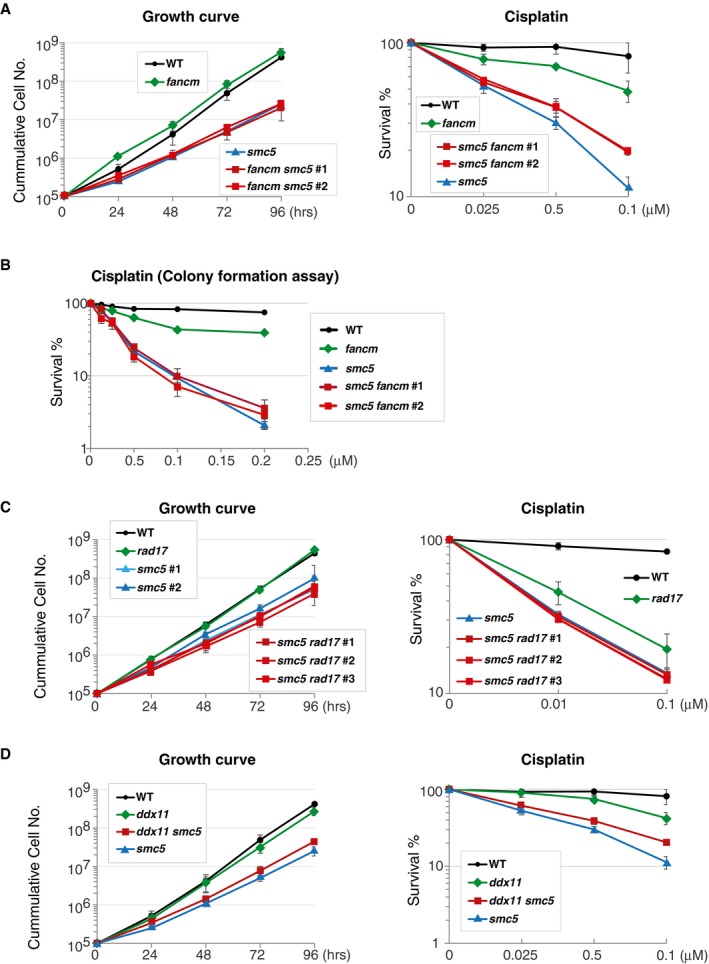
SMC5 acts jointly with FANCM and the FA‐like pathway involving the RAD17 checkpoint clamp loader and the DDX11 DNA helicase ALeft panel: Growth curve was carried out to test *smc5 fancm* cell proliferation. Data represent means ± SD of three experiments. Right panel: survival curve of *smc5 fancm* cells treated with different concentrations of cisplatin. Data represent means ± SD of the three experiments.BClonogenic assay to test cisplatin sensitivity of *smc5 fancm* cells in long‐term assays. Data represent means ± individual values of two independent experiments with two independent clones analyzed for *smc5 fancm*.C, DLeft panel: Growth curve was carried out to test *smc5 rad17* (C) and *smc5 ddx11* (D) cell proliferation. Data represent means ± SD of three experiments. Right panel: survival curve of *smc5 rad17* (C) and *smc5 ddx11* (D) cells treated with different concentrations of cisplatin. Data represent means ± SD of three experiments. Source data are available online for this figure.

We established double mutants between *fancm* and *smc5* knockouts and found these cells to be proliferating similarly with the *smc5* single mutants (Fig 3A). In regard to cisplatin sensitivity measured again by the Celltiter‐Glo assay, *smc5* mutants are more sensitive than *fancm*, while *smc5 fancm* mutants showed reduced cisplatin sensitivity compared with *smc5* (Fig 3A), a situation recapitulating the phenotype observed with *fancc* (Fig 2D), but in a situation in which *smc5* and *smc5 fancm* mutants proliferated equally well (although worse than WT). Moreover, long‐term clonogenic assays revealed a similar epistatic relationship between *fancm* and *smc5* mutations in regard to cisplatin sensitivity (Fig 3B).

**Figure EV3:**
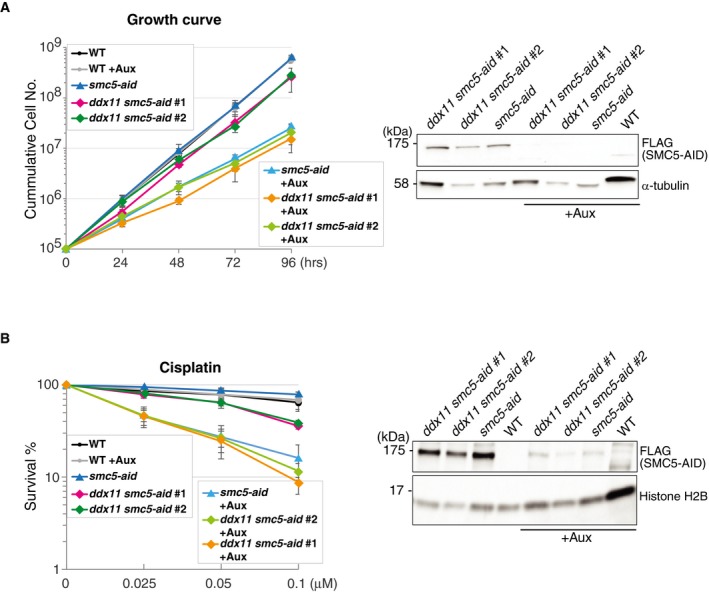
SMC5 functions jointly with DDX11 to promote DNA repair Left panel: Growth curve was carried out to test *ddx11 smc5‐aid* cell proliferation after SMC5‐AID depletion induced by auxin treatment. Data represent means ± SD of three experiments. Experiments were carried out at 39.5°C. Right panel: Western blot monitoring SMC5‐AID‐FLAG protein level in the presence or absence of auxin. Tubulin was used as loading control.Survival curve of *ddx11 smc5‐aid* cells treated with different concentrations of cisplatin after SMC5 depletion upon auxin treatment. Data represent means ± SD of four experiments. Right panel: Western blot monitoring SMC5‐AID‐FLAG protein level in the presence or absence of auxin. Histone H2B was used as loading control.

A backup pathway for the FA pathway involves the conserved DDX11 helicase and the checkpoint clamp loader RAD17, which act jointly to facilitate recombination‐mediated repair of bulky lesions in vertebrate cells 19, 20. In human cells, RAD17 also affects FANCD2 ubiquitylation 48. To study the relationship between this FA‐related pathway and SMC5 function, we established double mutants between *smc5* (and *smc5‐aid*) and *rad17, ddx11* mutations. The double mutants proliferated similarly with *smc5* single mutants, and both *rad17* and *ddx11* showed epistasis to *smc5* mutants toward cisplatin (Fig 3C and D). *ddx11* showed similar non‐additive relationship with depletion of SMC5‐AID in regard to proliferation and repair (Fig EV3A and B). Thus, SMC5 functions jointly with FANCM and FA‐related pathway involving DDX11 and RAD17 in DNA repair.

### SMC5/6 acts jointly with FANCD2 to mediate DNA repair and prevent genomic instability in human cells

**Figure 4:**
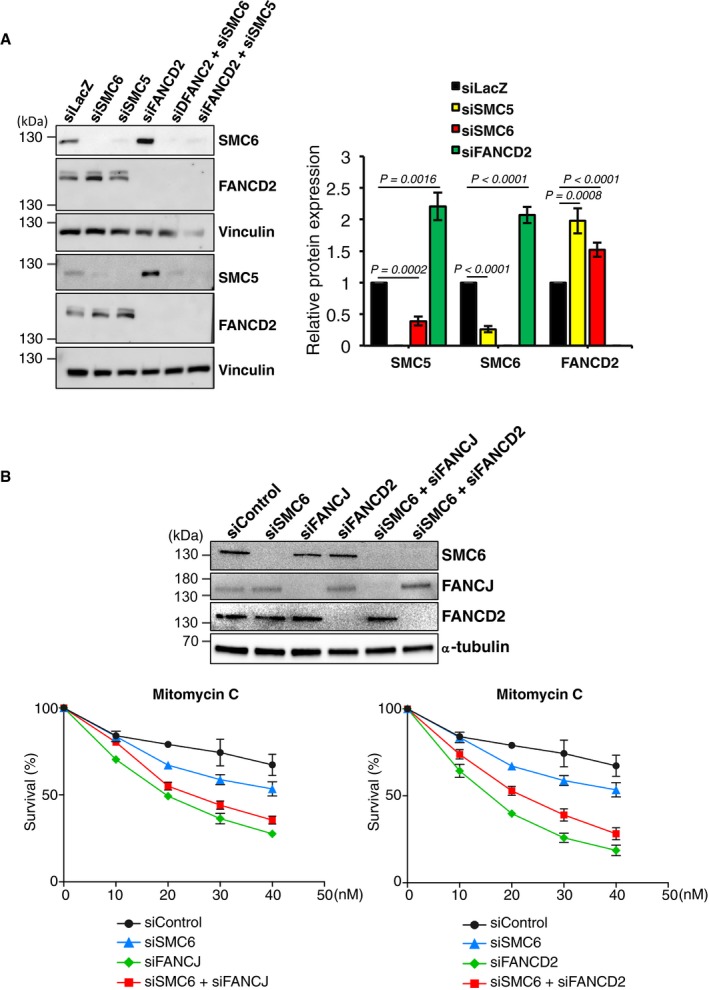
SMC5/6 and FANCD2 jointly promote DNA repair of MMC‐induced lesions Left panel: representative Western blots illustrating the expression of SMC6, SMC5, and FANCD2 in protein extracts from HeLa cells 48 h after transfection with the indicated siRNAs. Right panel: relative expression of SMC5, SMC6, and FANCD2. We performed individual experiments for each cell line with technical replicates. In each individual experiment, we first normalized SMC5, SMC6, or FANCD2 expression to that of Vinculin (internal control) and calculated its relative level in each cell line in relation to the target protein/Vinculin ratio of the siLacZ‐transfected cells, which was set as one in each experiment. Data present the mean ± SEM and were analyzed by unpaired *t*‐test. SMC5 in siSMC6: *n* = 7 and *P* = 0.0002; SMC5 in siFANCD2: *n* = 7 and *P* = 0.0016; SMC6 in siSMC5: *n* = 8 and *P* < 0.0001; SMC6 in siFANCD2: *n* = 24 and *P* < 0.0001; FANCD2 in siSMC6: *n* = 30 and *P* < 0.0001; and FANCD2 in siSMC5: *n* = 10 and *P* = 0.0008.Top: representative Western blots illustrating the expression of SMC6, FANCJ, and FANCD2 in protein extracts from HeLa cells 48 h after transfection with the indicated siRNAs. Bottom: survival curves of cells transfected with indicated siRNA and treated with different concentrations of mitomycin C (MMC). Data represent the means and SEM (standard error of the mean) of three independent experiments. Source data are available online for this figure.

To validate and extend our previous observations made in the chicken DT40 cell system, we analyzed the consequence of SMC5/6 depletion in HeLa cells. siSMC5 depletion and siSMC6 depletion were very efficient by 48 h post‐transfection and validated by using different siRNA sequences to avoid off‐target consequences of the siRNA approach. SMC5 or SMC6 depletion strongly affected the stability of the other partner (Fig 4A). Notably, their depletion was associated with an increased level of FANCD2, and conversely, FANCD2 depletion was associated with increased SMC5/SMC6 levels (Fig 4A).

**Figure EV4:**
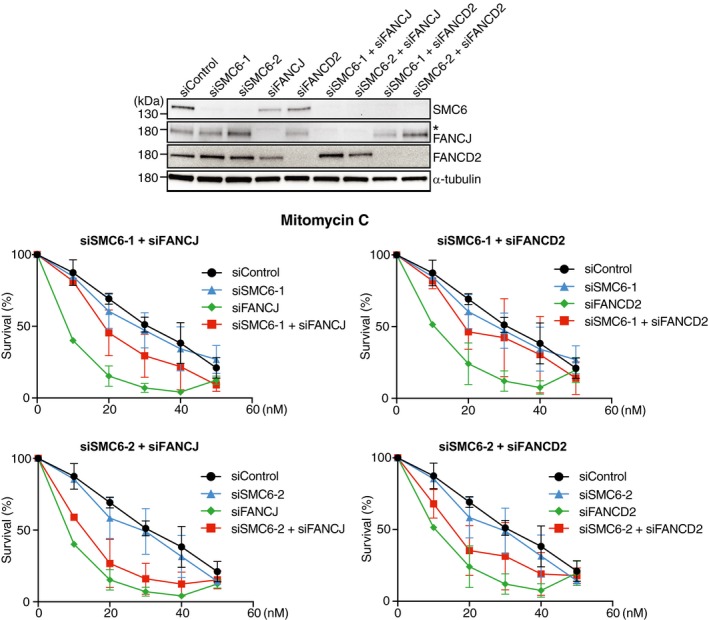
SMC5/6 does not affect FANCD2 focus formation Left panel: representative Western blots illustrating the expression of SMC5, SMC6, and monoubiquitylated FANCD2 in protein extracts from HeLa cells transfected with the indicated siRNAs and treated 48 h after transfection with aphidicolin 0.3 μM for 12 h. Vinculin is used as loading control. Right panel: representative spreads with FANCD2 foci and DAPI staining in control and SMC5/6‐depleted cells. The scale bar is 10 μm.

Cell survival in response to mitomycin C (MMC) exposure, measured using crystal violet staining, revealed that SMC6‐depleted HeLa cells are slightly more sensitive than control cells but significantly less sensitive than FANCD2‐ or FANCJ‐depleted cells. The simultaneous depletion of SMC6 and FANCJ had minimal effect on the MMC sensitivity of FANCJ‐depleted cells, whereas the downregulation of SMC6 in FANCD2‐depleted cells increased their resistance to MMC (Fig 4B). These results were obtained using smart siRNA pools, but qualitatively similar results were obtained using individual different siRNAs for SMC6 and a single siRNA for FANCD2 and FANCJ (Fig EV4). Thus, also in human cells, SMC6 acts in a DNA repair pathway non‐redundant with the one mediated by the FA proteins FANCJ and FANCD2.

**Figure 5:**
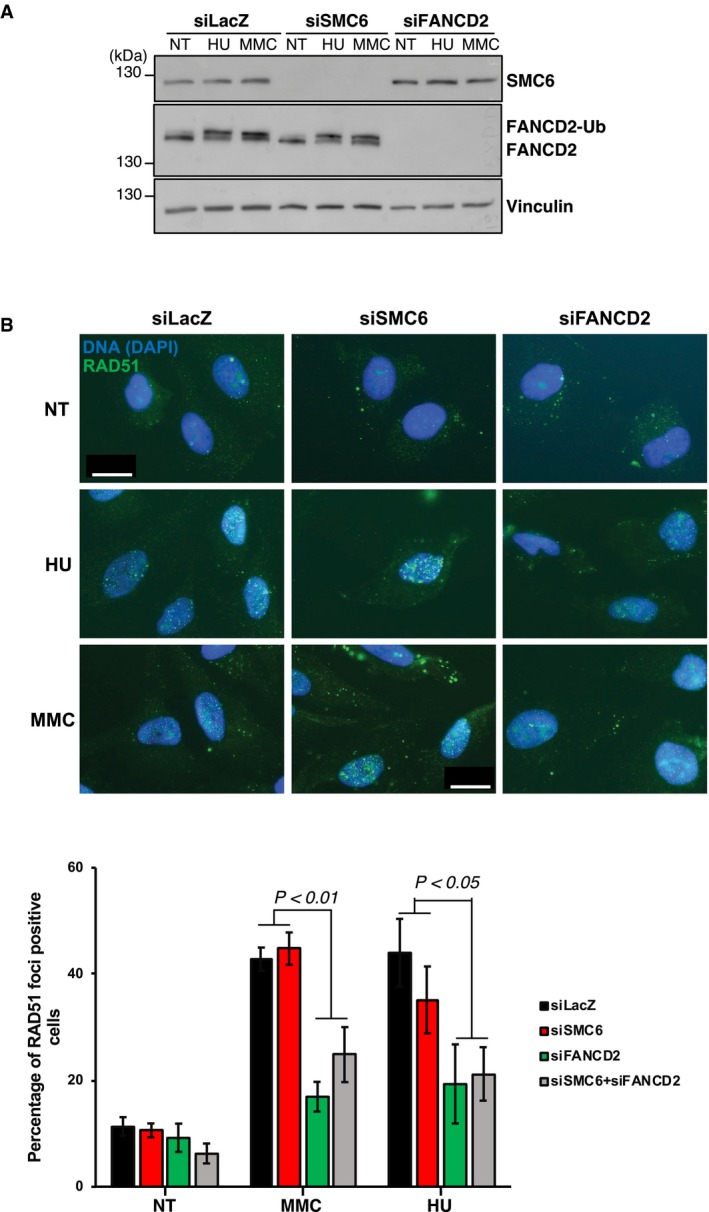
SMC6 is not critical for efficient FANCD2 ubiquitylation or RAD51 focus formation Representative Western blots illustrating the expression of SMC6 and of monoubiquitylated FANCD2 in protein extracts from HeLa cells transfected with the indicated siRNAs and treated 48 h after transfection with HU (5 mM, 18 h) or mitomycin C (200 ng/ml, 18 h). Vinculin is used as loading control.Top: representative images of nuclei (DAPI‐stained, blue) with RAD51 (green) foci in HeLa cells transfected with the indicated siRNAs and treated with HU or MMC, as indicated in panel (A). The scale bar represents 10 μm. Bottom: histograms presenting the percentage of RAD51 foci‐positive cells as evaluated in the different conditions of above. Data represent the means ± SEM and were analyzed by unpaired *t*‐test. siSMC6 vs. siLacZ: *n* = 3, NT, MMC or HU *P* = n.s.; siFANCD2 vs. siLacZ: *n* = 4, NT *P* = n.s., MMC *P* = 0.0003, HU *P* = 0.0479; and siSMC6 + siFANCD2 vs. siLacZ: *n* = 3, NT *P* = n.s., MMC *P* = 0.0439, HU *P* = 0.0458. Source data are available online for this figure.

**Figure EV5:**
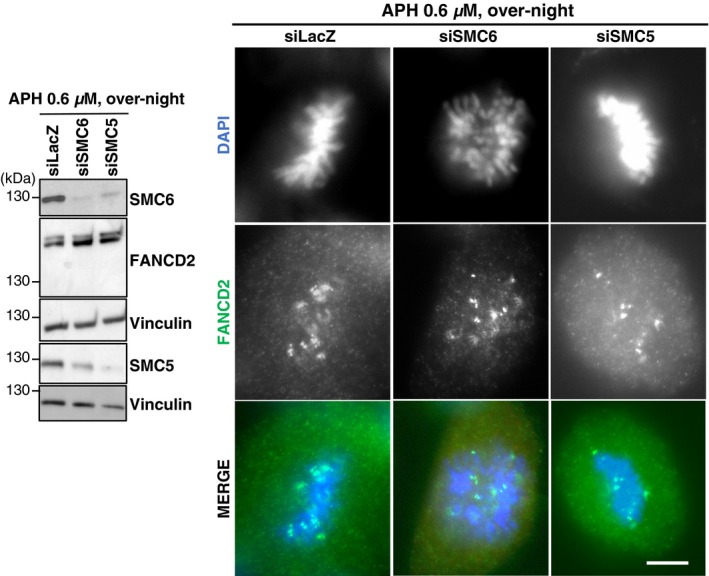
SMC5/6 does not affect FANCD2 foci formation Left panel: representative Western blots illustrating the expression of SMC5, SMC6, and monoubiquitylated FANCD2 in protein extracts from HeLa cells transfected with the indicated siRNAs and treated 48 h after transfection with aphidicolin 0.6 μM overnight. Vinculin was used as loading control. Right panel: representative spreads with FANCD2 foci and DAPI staining in control and SMC6‐depleted cells. The scale bar is 10 μm.

SMC6 depletion using different siRNAs did not affect FANCD2 monoubiquitylation in either untreated or replication‐stress conditions involving hydroxyurea (HU), MMC, or aphidicolin (APH) treatment (Figs 4A and 5A, and EV5) nor FANCD2 foci assembling in S/G2 phases of the cell cycle (Fig EV5), supporting the notion that SMC5/6 acts independently or downstream of FANCD2 activation.

The FANCD2 monoubiquitylation is important for optimal RAD51 focus formation 49, 50, 51, and *DDX11* deletion in DT40 that does not affect FANCD2 monoubiquitylation also reduces RAD51 focus formation 19. We assessed the consequences of SMC6 depletion on the formation of RAD51 foci in MMC‐ or HU‐treated HeLa cells. Differently from FANCD2 depletion, siSMC6 did not alter RAD51 foci *per se* and did not modify the frequency of RAD51 foci‐positive cells observed in the absence of FANCD2 (Fig 5B). Thus, our data suggest that SMC5/6 acts independently or downstream of RAD51 focus assembly, and downstream of FANCD2 and DDX11 that facilitate efficient RAD51 focus assembly.

**Figure 6:**
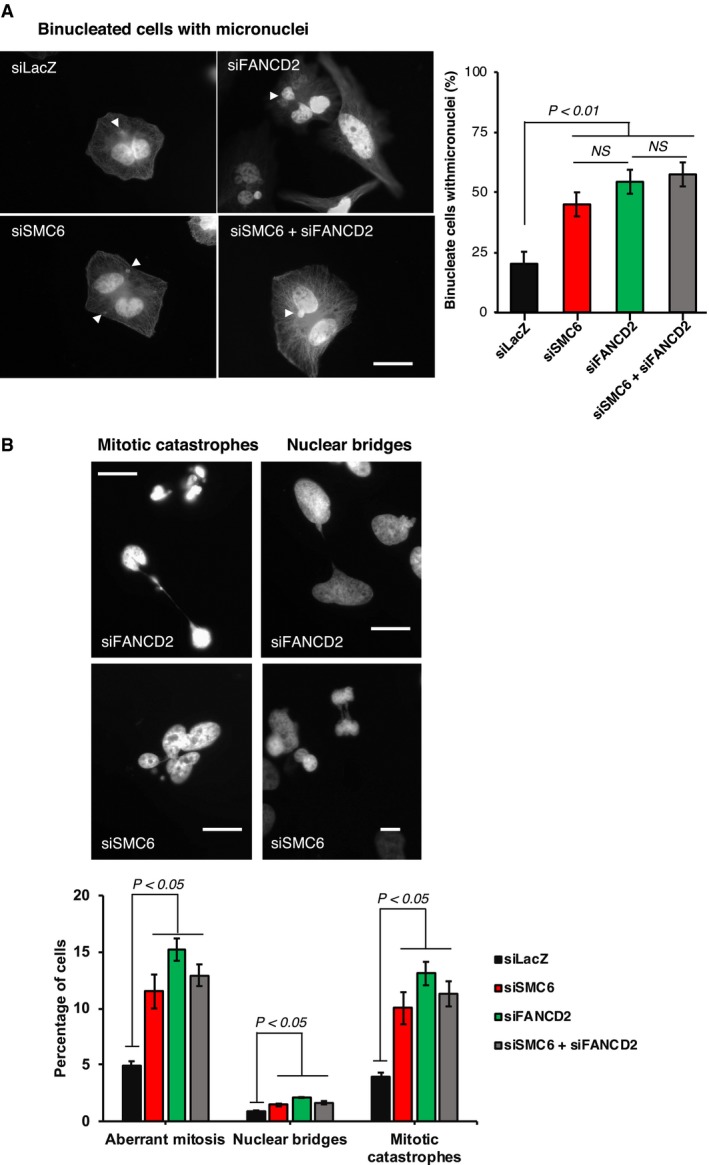
SMC6 acts jointly with FANCD2 to prevent micronuclei and mitotic abnormalities Left panel: representative images of anaphase HeLa cells with micronuclei (white arrows). Forty‐eight hours after transfection with indicated siRNAs, cells were incubated 18 h with 2 μg/ml cytochalasin B to block cytokinesis. The scale bar represents 20 μm. Right panel: histograms presenting the percentage of binucleate cells with micronuclei. Data represent the mean ± SEM of three independent experiments, *t*‐test, unpaired. siSMC6 vs. siLacZ, *P* = 0.0085; siFANCD2 vs. siLacZ, *P* = 0.0057; and siSMC6 + siFANCD2 vs. siLacZ, *P* = 0.0019.Top: examples of HeLa mitotic catastrophes and anaphase bridges as observed 48 h after siRNA transfection. The scale bar represents 20 μm. Bottom: histograms presenting the percentage of cells with mitotic abnormalities. Data represent the mean ± SEM of three independent experiments. *t*‐test, unpaired. siSMC6 vs. siLacZ: aberrant mitosis *P* = 0.0125, nuclear bridges *P* = 0.0198, mitotic catastrophes *P* = 0.0145; siFANCD2 vs. siLacZ: aberrant mitosis *P* = 0.0005, nuclear bridges *P* = 0.0002, mitotic catastrophes *P* = 0.0011; and siSMC6 + siFANCD2 vs. siLacZ: aberrant mitosis *P* = 0.0012, nuclear bridges *P* = 0.0116, mitotic catastrophes *P* = 0.0033. Source data are available online for this figure.

It was proposed that FANCD2 foci assembled during S phase at stalled/delayed replication forks persist into G2/M and up to the end of mitosis to rescue under‐replicated or untangled replicated regions, with this action serving to avoid anaphase bridges and genomic instability in daughter cells 21. Indeed, FANCD2 loss of function is associated with an increased frequency of mitotic catastrophes and nuclear bridges at anaphase–telophase as well as with increased frequency of post‐mitotic cells presenting micronuclei 21. Notably, SMC6‐depleted cells revealed a similar level of mitotic abnormalities with FANCD2‐depleted cells, and the frequency of these abnormalities remains unchanged in SMC6 and FANCD2 double‐depleted cells (Fig 6A and B). The results thus suggest joint action between SMC6 and FANCD2 in preventing genomic instability in mitotic and post‐mitotic cells.

### SMC5/6 physically interacts with FANCD2‐I in human cells

**Figure 7:**
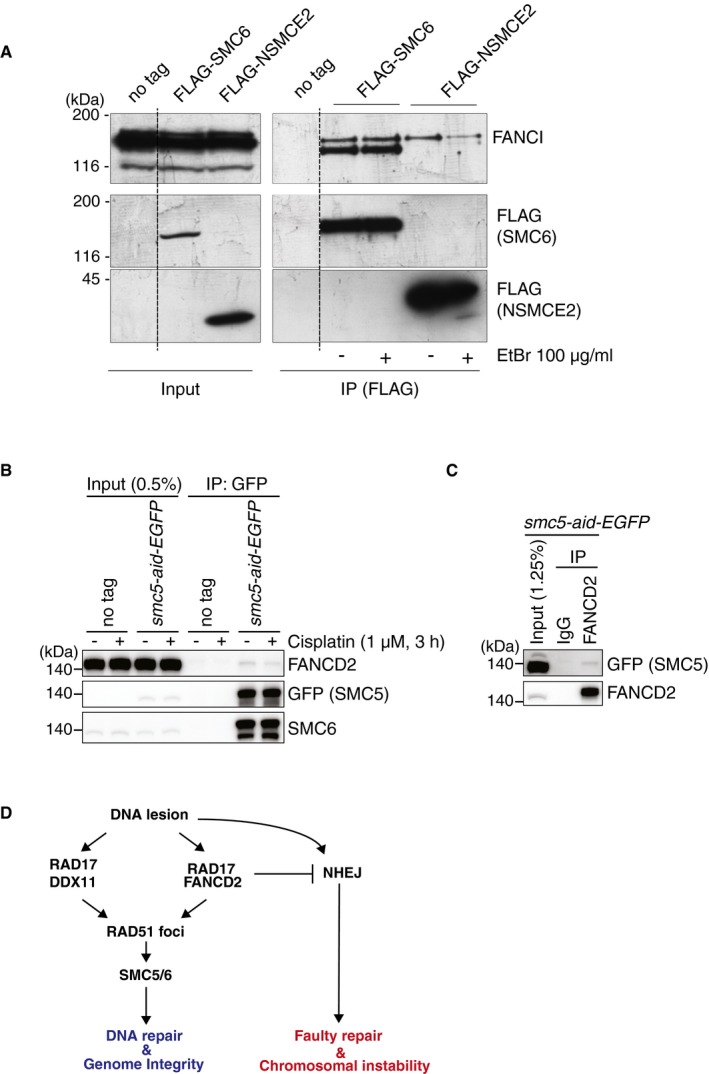
SMC5/6 physically interacts with FANCD2‐I in human cells AImmunoprecipitation of FLAG–SMC6 and FLAG–NSMCE2 with FANCI in HEK293T cells overexpressing the SMC6 or NSMCE2, respectively, in the presence or absence of ethidium bromide (EtBr). The experiment was performed three times, and the blot shows a representative experiment. The band under the one indicated as FANCI may represent a degradation product.B, CCo‐immunoprecipitation of endogenous SMC5‐mAID‐EGFP and FANCD2 in TK6 cells in the presence of benzonase. Both experiments were performed twice independently, with the note that the cisplatin condition was performed only in one of the experiments, and the FANCD2 pull‐down in the experiment not shown was performed without benzonase.DModel schematizing the proposed view of SMC5/6 action in genome integrity and DNA repair. SMC5/6 acts differently from non‐homologous end‐joining and downstream of RAD51 filament formation and of homologous recombination branches mediated by DDX11, RAD17, and FANCD2, RAD17. Source data are available online for this figure.

To examine potentially informative physical interactions, we transiently expressed FLAG‐tagged SMC5, SMC6, and NSMCE2 in HEK293 cells and immunoprecipitated the complexes with an anti‐FLAG antibody. Mass spectrometry identified the other SMC5/6 components in the immunoprecipitate of SMC5, along with a few other proteins of interest, among which FANCI (Table 1). Immunoblotting further validated that FANCI was present in the immunoprecipitates of SMC5/6 and NSMCE2 (Fig 7A), even in the presence of ethidium bromide (EtBr), which binds DNA and disrupts DNA–protein interactions.

**Table 1:** FLAG‐SMC5 IP‐MS

Protein ID	Peptide number^a^
SMC5	119.5
NSMCE3	21
NSMCE1	14
NSMCE4A	11
SMC6	39
NSMCE2	9
TUFT1	6.5
MLF2	1.5
CPD	1.5
FANCI	1

a The numbers of peptides are averaged over two technical replicates.

Because in the above experiments SMC5/6 and NSMCE2 were overexpressed, we examined whether the interaction was observed among endogenous proteins. As we experienced low IP efficiency with commercial SMC5 antibodies, we tagged endogenous SMC5 alleles C‐terminally with the mAID‐EGFP tag in human TK6 cells using CRISPR‐Cas9, using the strategy reported in Ref. 52. Using SMC5‐mAID‐EGFP TK6 cells, we found that SMC5‐EGFP pull‐down led to co‐immunoprecipitation of FANCD2, and vice versa, independently of induced DNA damage (Fig 7B and C). These results suggest that SMC5/6 physically interacts with FANCD2‐I *in vivo*.

## Discussion

Studies of the Smc5/6 complex in budding and fission yeast are consistent with the involvement of this complex in dynamic DNA processes, such as stabilization of stalled replication forks and regulation of recombination intermediate resolution 9, 10, 11, 13. The essential functions of the SMC5/6 complex, at least in budding yeast, primarily manifest after the bulk of replication is complete, and involve processes mediating replication termination and DNA synthesis through difficult to replicate regions 11, 13, such as ribosomal DNA, rDNA 12, 53. The postreplicative or late replication nature of the essential processes requiring SMC5/6 suggests that this complex may be especially important during late replication, in a subset of DNA metabolism reactions with repercussions on the mitotic chromosome structure and its compaction 54. In budding yeast, those processes may be particularly relevant to rDNA condensation and segregation 12, whereas in vertebrates they may encompass several classes of fragile sites or structures that remain to be identified. Nevertheless, the partners of SMC5/6 in these repair processes remain poorly understood, especially in vertebrate cells.

In this study, we undertook a dual approach to better understand the functions of vertebrate/mammalian SMC5/6 in DNA repair and genome stability. One approach aimed at identifying new interacting partners of SMC5/6 in mammalian cells, while the other addressed the molecular functions of SMC5/6 in DNA repair and genome integrity by examining several mutant combinations in DT40 and upon knockdown in HeLa cells. Remarkably, both our approaches intersected on the FA pathway and particularly FANCD2‐I, already reported to play roles in facilitating replication through common fragile sites (CFSs) 21, 55, 56, 57 and for preventing fragile site expression in the presence or absence of replication stress 45, 58, 59, 60. Here, we find that SMC5/6 components interact physically with FANCD2‐I and function jointly with FA proteins (FANCC, FANCM, FANCJ, FANCD2) and FA‐related pathways (RAD17, DDX11) in the repair of ICLs created by cisplatin and mitomycin C. Considering that SMC5/6 depletion does not impair FANCD2 ubiquitylation and either FANCD2 or RAD51 focus formation, these results indicate roles of SMC5/6 in the late steps of HR, perhaps by resolving emerging recombination intermediates (Fig 7D). This could explain the effects of SMC5/6 depletion on anaphase bridge and micronuclei formation, which resemble in extent and are non‐additive with the ones of FANCD2 depletion. FANCD2 counteracts NHEJ, and inhibition of NHEJ in FA rescues survival and genetic instability in FA 33, 34. Our new results suggest that this rescue happens because cells become able to funnel lesions in the HR pathway dependent on DDX11 and SMC5/6 (Fig 7D). Because ICL repair resembles replication termination 61 and termination regions are predisposed to fragility 62, 63 and protected by budding yeast Smc5/6 13, we propose joint roles of SMC5/6 and FANCD2‐I at these regions and possibly at other subsets of CFSs.

Our findings can be accommodated by a model envisaging two functional interaction points between the FA pathway and SMC5/6: one in which SMC5/6 function/recruitment to the DNA lesion or terminal sites of replication is facilitated by FA components such as FANCM, and a subsequent one in which SMC5/6 facilitates the repair function of the FA pathway (Fig 7D). The role of SMC5/6 in facilitating DNA repair may be manifested via recruitment and/or Sumoylation of BLM, already reported to act jointly with FANCD2 and SMC5/6/NSMCE2 in certain conditions of replication stress 21, 64. Alternatively, SMC5/6 may downregulate the fork transversal function of FANCM and/or activate the degradation/mobilization of other replication, repair, or structural factors to facilitate lesion bypass and subsequently replication completion. This latter envisaged function may involve the ubiquitin and SUMO ligase activity of the SMC5/6 complex and potentially facilitate replisome disassembly during replication termination 65 or cohesin removal to facilitate repair completion and normal chromosome structure in prophase 66. We thus envisage that there remain undiscovered genetic disorders caused by mutations in genes of the *SMC5*/*6* complex that lead to FA or FA‐like disorders and in which the physiological role SMC5/6 in mediating DNA repair and replication termination becomes more apparent.

## Materials and Methods

### Cell lines and genotoxic treatments

All DT40 cell lines used in the study were derived from the DT40 WT clone 18 67 and reported in Table 2.

**Table 2:** DT40 cell lines used in the study

Code	Clones	Description	Origin
FR#1	WT	DT40 cl. 18 (WT)	Laboratory stock
FR#2	*smc5#1*	WT, *smc5::*Puro	This study
FR#4	*smc5#2*	WT, *smc5::*Puro	This study
FR#3	*smc5#3*	WT, *smc5::*Puro	This study
#FR29	*rad17#*	WT, *rad17::*His	Takuya Abe, Laboratory stock
FR#42	*smc5 ddx11#1*	*smc5*#2, *ddx11::*Hygro, Bsr	This study
FR#48	*smc5 fancc#1*	*smc5*#2, *fancc::*His	This study
FR#49	*smc5 fancc#2*	*smc5*#2, *fancc::*His	This study
FR#50	*fancc#*	WT, *fancc::*His	This study
FR#24	*smc5‐aid#1*	WT, TIR1‐9MYC*::*His, SMC5‐3AID‐6FLAG*::*Bsr	This study
FR#20	*smc5compl#1*	*smc5#1,* SMC5‐HA*::*Bsr	This study
FR#21	*smc5compl#2*	*smc5#1,* SMC5‐HA*::*Bsr	This study
FR#22	*smc5#1 *+* mock*	*smc5#1*, mock*::*Bsr	This study
#35	*ddx11#1*	WT, *ddx11*::Bsr, Puro	Takuya Abe, Laboratory stock
FR#9	*smc5 rad17#1*	*smc5#2, rad17::*His	This study
FR#10	*smc5 rad17#2*	*smc5#2, rad17::*His	This study
FR#11	*smc5 rad17#3*	*smc5#2, rad17::*His	This study
FR#44	*smc5 fancm#1*	*smc5#2, fancm::*His, Bsr, Puro	This study
FR#35	*smc5 fancm#2*	*smc5#2, fancm::*His, Bsr, Puro	This study
FR#46	*fancm#1*	WT, *fancm*: His, Bsr	This study
*wtM*	wtM	DT40 cl. 18 (WT)	Dr. Morrison laboratory stock
N29	*smc5N29*	WT, *smc5*	18, Dr. Morrison laboratory stock
K30	*smc5 ku70#*	*ku70, smc5off*	18, Dr. Morrison laboratory stock
#78	*Ku70#*	WT, *ku70*	18, Dr. Morrison laboratory stock
R379	*SMC5‐HA*	WT, SMC5‐HA::Eco	This study

HeLa cells were purchased from ATCC and routinely maintained in 75‐cm^2^ flasks in DMEM (Gibco^®^) + 13% fetal calf serum (Gibco^®^) + 1% penicillin–streptomycin (Gibco^®^) with reseeding every 4 days after trypsinization.

TK6 cells expressing *TIR1* are a gift from Shunichi Takeda at Kyoto University. TK6 cells were cultured in RPMI 1640 (Cat. No. BE12‐167F, Lonza), horse serum 5% (Cat. No. 16050‐122, Life Technologies), l‐glutamine 2 mM (Cat. No. X0550, Microtech or Euroclone, Cat No. LOBE17605F), sodium pyruvate 1.8 mM (Cat. No. L0642, Microtech), and P/S (Cat. No. L0022, Microtech or Euroclone, Cat. No. ECB3001L), at 37°C.

Genotoxic treatments were performed using mitomycin C (MMC, Cat. No. M0503, Sigma‐Aldrich), cisplatin (CDDP, Cat. No. P4394, Sigma‐Aldrich), hydroxyurea (HU, Cat. No. 400046, Sigma‐Aldrich), aphidicolin (APH, Cat. No. A0781, Sigma‐Aldrich), or formaldehyde (Cat. No. F8775, Sigma‐Aldrich) at the indicated concentrations and time periods for chronic and acute treatments.

### DNA restriction enzymes and DNA ligase

All restriction enzymes and T4 DNA ligase were purchased from New England Biolabs (Frankfurt, Germany) and used according to the manufacturer's specification. GeneArt^®^ Seamless Cloning and Assembly enzyme mix (Cat. No. A14606, Thermo Fisher Scientific) and GeneArt^®^ Seamless Plus Cloning and Assembly enzyme mix (Cat. No. A14603, Thermo Fisher Scientific) were used to clone the homologous recombination arms for SMC5 C‐terminus tagging in DT40 cells.

### Plasmids

The vectors used for DT40 cloning were previously reported 68, 69. Vectors used for SMC5 tagging with HA and AID were modified in the laboratory starting from a backbone received from the Hirota Lab 25. The Px458 CRISPR/Cas9 vector reported in Ref. 70 was used in this study to establish *fancc*,* and fancm* knockouts in WT and *smc5* backgrounds. Guide DNA targeting for *fancc* and *fancm* was purchased from Sigma‐Aldrich and cloned in the px458 vector (https://www.addgene.org/48138/), generating vectors FR18# (for *fancc*) and FR20# (for *fancm*), respectively. FR18# was transiently co‐transfected with vector #122 71 in *smc5* and WT, respectively, in order to induce double‐strand breaks to enhance HR and the insertion of the resistance marker contained in the vector in order to KO *fancc*. FR20# was transiently co‐transfected with vectors #12–31 and #12–29 in *smc5* or WT in order to induce double‐strand breaks to enhance HR and the insertion of the resistance marker contained in vector in order to KO *fancm*.

SMC5‐3xmAID‐6xFLAG Flip‐In was generated from genomic PCR products combined with p3xmAID‐6xFLAG containing histidinol D selection marker cassette. Genomic DNA sequences were amplified using primers 5′‐ATAAAAGTCGACGGAGAGCTTAATTTTGTGTGAC‐3′ (containing SalI restriction enzyme site) and 5′‐ACCATTGCTAGCCTGTTCATCCATTCTTCC‐3′ (containing *NheI* restriction enzyme site). Amplified PCR product was purified by gel extraction and digested by *SalI* high‐fidelity and *NheI* high‐fidelity restriction enzymes, then purified again, and ligated in p9xMyc vector 72. Then, they were cloned with the same strategy in 3xmAID‐6xFLAG vector 24. The Flip‐In vector was then linearized at one restriction enzyme site in the middle of the homology region and transfected to DT40 cells as previously described 25.

SMC5‐HA Flip‐In was generated from genomic PCR products combined with p3xmAID‐6xFLAG containing Ecogpt selection marker cassette. Genomic DNA sequences were amplified using primers 5′‐ATAAAAGTCGACGGAGAGCTTAATTTTGTGTGAC‐3′ (containing SalI restriction enzyme site) and 5′‐ATGCGGCGCGCCTCAAGCGTAATCTGGAACGTCATATGGATACTGTTCATCCATTCTTCCAAGTCGC‐3′ (containing sequence coding HA‐tag and *AscI* restriction enzyme site). Amplified PCR product was digested by *SalI* and *AscI* restriction enzymes and purified by gel extraction, and then ligated into p3xmAID‐6xFLAG vector. The Flip‐In vector was then linearized by *SnaBI* restriction enzyme before transfection.

SMC5 knockout construct was generated by modifying the SMC5 KO‐Neo vector published in Ref. 18, which was digested with *BamHI* in order to cut exon 1 from the 5′ arm, purified by gel extraction, and ligated with loxp (histidinol D) selection marker cassette. Then, the vector was digested with *PvuI* and *BamHI* restriction enzymes, purified by gel extraction, and ligated with loxP (puromycin) selection marker cassette. SMC5‐KO‐Puro vector was then linearized with *NotI* before being transfected to DT40 cells. *rad17* and *ddx11* mutations were established as previously reported 19.

To construct the targeting vectors for introducing mAID‐EGFP tag to the C‐terminus of the *SMC5* gene in TK6 cells, the 5′ arm was amplified by using primers 5′‐AGGGCGAATTGGAGCTCCCCCAGTAGATAGTCTTGTGGAATAGTCAC‐3′ and 5′‐TTGGCGCCTGCACCGGATCCAGAAGGTTGAGTGAATGTAATACg‐3′. The 3′ arm was amplified by using primers 5′‐CGAAGTTATTAGGTCCCTCGTGGAAACTATAATGACCTTTCC‐3′ and 5′‐GGGAACAAAAGCTGGGGAACCTCTTCTGTTCAAAGACATGACTTTGG‐3′. (Primers contain homology sequence to the backbone vectors.)

The 5′ and 3′ arms were assembled with pBS‐mAID‐GFP‐loxP‐Neo (neomycin‐resistant cassette) or Hyg (hygromycin‐resistant cassette) 52 digested with *EcoNI* and *SmaI* using GeneArt™ Seamless PLUS Cloning and Assembly Kit (Cat# 14603, Thermo Fisher Scientific) according to the manufacturer's protocol. To construct the CRISPR/Cas9 vector for targeting *SMC5*, annealed primers 5′ CACCGTATGGCTCAACTGAATAAA‐3′ and 5′‐AAACTTTATTCAGTTGAGCCATAC‐3′ were inserted into the *BbsI* site of pX458 vector (Cat# 48138, Addgene). The two resulting targeting vectors containing Neo and Hyg antibiotic markers and the pX458‐gRNA vector were transfected into wild‐type expressing the *TIR1* gene.

TK6 cells expressing *TIR1* were electroporated using Neon^®^ Transfection System MPK5000 (voltage/width/number of pulses: 1,350 V/10 ms/3), with medium containing no PBS. Cells were selected 24 h after electroporation, using the drugs corresponding to the resistance marker(s) used, and then, the cells were plated in 96 wells to get single‐cell clonal population. Cells were grown in medium until a single colony/well was visible, then picked up with a filter tip, and moved to a 24‐well dish. Then, genomic DNA was extracted, the genotype was established by PCR, and ultimately confirmed by WB.

### Antibodies

As primary antibodies for DT40 cell extracts, we used the following: anti‐α‐tubulin, mouse monoclonal antibody, clone B‐5‐1‐2 (Cat. No. T5168, Sigma‐Aldrich), 1:5,000 for WB; anti‐CHK1 (G‐4), mouse monoclonal antibody (Cat. No. sc‐8408, Santa Cruz Biotechnology), 1:1,000 for WB; anti‐CHK1‐P S345, rabbit polyclonal antibody (Cat. No. 133D3, #2341, Cell Signaling), 1:1,000 for WB; anti‐FANCD2, rabbit polyclonal antibody, the Takata laboratory, Kyoto medical University 20, 1:4,000 in BSA 5% TBS‐T 0.1% for WB; anti‐Flag M2, mouse monoclonal Ab (Cat. No. F1365 Sigma‐Aldrich), 1:3,000 for WB; anti‐Histone H2B, rabbit polyclonal (Cat. No. ab1790, Abcam), 1:3,000 for WB; anti‐HA, rat monoclonal (3F10, Cat. No. 11867423001, Roche), 1:500 for WB; and anti‐BrdU antibody, mouse monoclonal (B44, Cat. No. 5295722, BW), 1:5 for FACS. For WB on HeLa cell extracts, the following antibodies were used: anti‐SMC5, mouse monoclonal antibody, clone B11, 1/250 (Cat. No. sc‐393282, Santa Cruz Biotechnology); anti‐SMC6 mouse monoclonal antibody, clone A3, 1/250 (Cat. No. sc‐365742 Santa Cruz Biotechnology); anti‐FANCD2 mouse monoclonal antibody, clone FI17, 1/500 (Cat. No. sc‐20022, Santa Cruz Biotechnology); anti‐Vinculin mouse monoclonal [SPM227], 1/3,000 (Cat. No. ab18058, Abcam); and anti‐FANCJ rabbit polyclonal antibody, 1/1,000 (Cat No. NB100‐416, Novus Biologicals). For Co‐IP of SMC5, SMC6, NSMCE2, and FANCI, an antibody against FANCI home‐made by Weidong Wang's Lab at NIH/NIA was used. For Co‐IP in TK6 cells, anti‐GFP rabbit polyclonal (Cat. No. TP401, OriGene) was used for both IP (2.5 μg/sample) and WB (1:1,000).

As secondary antibodies, the following were used: goat anti‐mouse secondary antibody, horseradish peroxidase‐conjugated (Cat. No. 170‐6516, Bio‐Rad), 1:5,000 for WB; goat anti‐rabbit secondary antibody, horseradish peroxidase‐conjugated (Cat. No. 170‐6515, Bio‐Rad), 1:5,000 for WB; and donkey anti‐mouse FITC (Cat. No. 11‐095‐150, Jackson ImmunoResearch), 1:50 for FACS. For experiments performed in HeLa cells, secondary anti‐mouse or anti‐rabbit antibodies were from Bethyl.

### Cell culture and transfection procedures

All DT40 cells were derived from DT40 WT clone 18 67. DT40 cells were cultured in DMEM F‐12 medium (Cat. No. 31331‐093, Life Technologies or Cat. No. L0090‐500, Biowest) supplemented with 10^−5^ M mercaptoethanol (Cat. No. 31350‐010, Life Technologies), 10% fetal bovine serum, South America (Cat. No. S1810‐500, Biowest), and 1% chicken serum (Cat. No. C5405‐100ML, Sigma‐Aldrich), and P/S (Cat. No. L0022, Microtech) and grown at 39.5°C if not otherwise indicated.

For drug selection during knockout/knock‐in generation, cells were selected 16–24 h after electroporation, using the drug corresponding to the employed resistance marker, and then the cells were plated in 96 wells to get single‐cell clonal population. Cells were grown in medium until a single colony per well was visible, then it was picked up with a filter tip, and moved to a 24‐well dish. Then, genomic DNA was extracted and the genotype was checked by PCR, and eventually, mRNA expression was checked with reverse transcriptase–PCR (RT–PCR) for the indicated clones. For DT40 cells, electroporation was carried out using Gene Pulser II^®^ Electroporation System (Cat. No. 165‐2105, Bio‐Rad Laboratories, Hercules, CA, USA). Methods for DNA transfection and selection of clones were performed as described previously 67.

HeLa cells were plated at 2 × 10^5^ cells/well in a 6‐well plate and then transfected 24 h later with a mix of 190 μl of Opti‐MEM^®^ (Invitrogen™), 8.5 μl of INTERFERin^®^ (Polyplus), and 2.2 μl of siRNA (20 nM final) incubated 10 min at RT before use. siLacZ transfection was used as a control.

**Table:** 

siSMC5‐a	5′‐GGAACUUCAGCAGGGCUUUAAUAGUA‐3′
siSMC5‐b	5′‐GGCAUUAUGUGAAGGCGAAAUAAUU‐3′
siSMC6‐a	5′‐GACCUAUCUUGAUCUGGAUAGUAAA‐3′
siSMC6‐b	5′‐CAAAUUCUUCAUGAAAGCAACGCAA‐3′
siFANCD2	5′‐GGAGAUUGAUGGUCUACUA‐3′
siFANCJ	5′‐ CAUACAGGGCCUUAAACCA‐3′
siLacZ	5′‐CGUCGACGGAAUACUUCGA‐3′

For the MMC sensitivity assays in HeLa presented in Fig 4B, cells were transfected with ON‐TARGETplus SMARTpool siRNAs (Dharmacon) targeting SMC6 (L‐018408‐01‐0010), FANCJ (L‐010587‐00‐0005), FANCD2 (L‐016376‐00‐0010), and siRNA control (non‐targeting pool) by using Lipofectamine RNAimax^®^ according to the manufacturer's protocol. Knockdown of proteins was analyzed by Western blot (WB). In addition, single siRNAs, siSMC6‐a, and siSMC6‐b with sequences indicated above were tested together with siRNA for FANCD2 and FANCJ (indicated above) in sensitivity assays shown in Fig EV4.

### Growth curve

For proliferation curves, cells were plated with a starting concentration of 10^5^ cells/ml in triplicate. They were counted at the indicated time points with the Burker chamber. Before counting, they were stained with erythrosine (1 volume of medium containing cells and 1 volume of erythrosine) to mark viable cells. Raw data were plotted in Excel to derive growth curves and to calculate the doubling time of individual cell lines.

### Auxin treatment

When indicated, auxin was added to the medium to a final concentration of 500 μM. Cells were treated with auxin for the indicated time, dependent on the assay. In order to check the depletion or the recovery of the expression of the target protein, 1 × 10^6^ cells were pelleted, then washed 1× with PBS 1×, and lysed with sample buffer 1×. Since auxin is reported to be degraded in the media, during proliferation experiments, auxin concentration was maintained by diluting the culture with fresh medium containing auxin. In Celltiter‐Glo ATP sensitivity assays, cells were supplemented with auxin after 24 h of treatment.

### Quantitative PCR for mRNA

Total RNA was isolated from DT40 cells by using TRIzol RNA Isolation Reagents (Invitrogen) according to the manufacture's protocol. cDNA library was prepared from total RNA by using SuperScript™ III Reverse Transcriptase (Invitrogen) according to the manufacture's protocol. Quantitative PCR was performed using GoTaq^®^ qPCR Master Mix (Promega) and LightCycler^®^ 96 Instrument (Roche) according to the manufacture's protocols. *ACTB* was used as house‐keeping gene. Used primers are described below.

ACTB fw: 5′‐CGTGCTGTGTTCCCATCTATCGTG‐3′
ACTB rv: 5′‐TACCTCTTTTGCTCTGGGCTTCATC‐3′
SMC5 fw: 5′‐GCAGGGCTGCTTGAAAGGTCTAGTG‐3′
SMC5 rv: 5′‐GCTTCCTGTTGATATGCCAGGTTGAC‐3′

### Drug sensitivity assays (Celltiter‐Glo, colony survival, and crystal violet assays)

Sensitivity assays in DT40 were carried out treating 2 × 10^4^ cells/ml with the indicated drug concentration for 48 h. To assess cellular sensitivity, we measured the amount of ATP levels using Celltiter‐Glo^®^ assay (Cat. No. G7571, Promega). Luminescence levels were detected using Victor3 TM (PerkinElmer). Control cells were treated with the vehicle diluted at the same concentration of the drug used in the assay. We have carried out each biological experiment in technical triplicates, and in certain occasions, eliminated outliers. We defined as outlier the measurement having a ratio bigger than 25% of each of the other two measurements and with their averages, dividing the smaller data with the bigger data. The other two data should not have a ratio bigger than 25% among themselves. Each experiment was conducted in at least three independent biological experiments.

To determine cisplatin sensitivity in DT40 by colony survival assay, an appropriate number of DT40 cells were inoculated in a medium supplemented with 1.5% (w/v) methylcellulose, 15% fetal bovine serum, and 1.5% chicken serum. Indicated concentration of cisplatin was added and mixed for 6 h before inoculating cells. Colonies were counted after 14 days, and the percent survival was determined relative to the number of colonies of untreated cells. Two independent biological experiments and two independent clones for *smc5 fancm* were used in the analysis shown in Fig 3B.

For cell viability assays in HeLa (Figs 4B and EV4), 2–3 × 10^3^ cells/well were seeded in 48‐well plates and allowed to adhere for 8–10 h. Chronic drug treatment was given to cells with indicated concentrations for 5 days. The cell viability was measured by staining the cells using 0.5% crystal violet containing 20% methanol, and the graph was plotted by normalizing with untreated cells. Three independent biological experiments were performed.

### SDS–PAGE and Western blot

DT40 cells were harvested by trypsin treatment and lysed or fractionated according to the experimental aim. Protein quantification was obtained using the Bradford reagent assay (Cat N., Bio‐Rad Laboratories, Hercules, CA, USA), according to the manufacturer's instructions. Samples were boiled in sample buffer (50 mM Tris–HCl pH 6.8, 10% glycerol, 1.6% SDS, 0.1 M DTT, 0.1% bromophenol blue). Whole‐cell lysates (WCLs) were separated on 4–12% Blot™ Bis‐Tris Plus (NW04125BOX, Thermo Fisher Scientific) or Criterion TGX Stain‐Free (#5678094, Bio‐Rad). Resolved samples were transferred to Hybond‐ECL nitrocellulose membrane (Amersham Bioscience, Glattbrugg, Switzerland), 1 h at 100 V or overnight at 30 V (4°C). Then, the membrane was saturated with 5% non‐fat milk Tris‐buffered saline (TBS 10 mM Tris–HCl PH 7.4, 100 mM NaCl) supplemented with 0.1% Tween‐20 (TBS‐T). The membranes were incubated with primary antibodies for 1 h at room temperature or overnight at 4°C. Then, the membranes were washed as mentioned above and incubated with the horseradish peroxidase (HRP)‐conjugated secondary antibody for 1 h at room temperature. The luminescent signal was detected by PICO or FEMTO reagent (Cat No. #34080, #34095, Thermo Fisher Scientific) on Amersham Hyperfilm™ ECL films (Cat No. 28906836 GE Healthcare UK, Little Chalfont, UK) or Chemidoc XRS+ System (Cat No. 1708265; Bio‐Rad Laboratories, Hercules, CA, USA). Western blot bands were analyzed by ImageJ software (Rasband, W.S., ImageJ, U.S. National Institutes of Health, Bethesda, MD, USA, http://rsb.info.nih.gov/ij/, 1997–2009) for film acquisitions.

### FACS analysis

Bidimensional FACS analysis was carried out pulsing the cells with BrdU, final concentration 20 μM for 15 min at 39.5°C (10 mM stock, Cat. No. B9285, Sigma‐Aldrich). After pelleting 1 × 10^6^ cells, cells were resuspended in 50 μl ice‐cold PBS, and then 1 ml of 70% ethanol (ice cold) was added. Cells were washed once in 1 ml PBS 1% BSA (4°C in all the steps if not differently indicated), then resuspended in 1 ml denaturing solution (2N HCl), and incubated at room temperature for 25 min.

Cells were washed twice with 1 ml PBS 1% BSA. Then, cells were stained with primary mouse anti‐BrdU antibody (B44, Cat. No. 5295722, BW), diluted 1:5 in PBS 1% BSA, and incubated 1 h at room temperature, light‐protected. They were washed once in 1 ml PBS 1% BSA, then the pellet was resuspended in 100 μl anti‐mouse secondary goat anti‐FITC antibody diluted 1:50 in PBS 1% BSA, and incubated for 1 h at room temperature, light‐protected (Cat. No. F0257, Sigma‐Aldrich). Cells were then washed once in 1 ml PBS 1% BSA, then resuspended in 1 ml propidium iodine F.C. 2.5 g/ml (50 μg/ml, Cat. No. P4170, Sigma‐Aldrich) containing RNase I F.C. 250 μg/ml, and incubated overnight at 4°C or 30 min at 37°C. Data were acquired using FACSCalibur (BD, Biosciences). Data analysis was carried out using Cell Quest.

Note: If not differently indicated, all the solutions were ice‐cold. Cells were spinned down at 300 × *g* at 4°C, for 5 min.

### Immunofluorescence

Cells were seeded at 2 × 10^5^ cells/well in a 6‐well plate on glass slides. After treatment, if any, cells were washed with PBS and then incubated in pre‐extraction buffer CSK100 (100 mM NaCl, 300 mM sucrose, 3 mM MgCl_2_, 1 mM EGTA, 0.2% Triton X‐100, PIPES pH 6.8 10 mM, anti‐protease) for 5 min at room temperature and then fixed for 10 min with PBS + 4% formaldehyde, and finally permeabilized with PBS + 0.1% Triton X‐100 for 10 min at RT. The cells were incubated 1 h with PBS + 5% BSA and then 1 h at 37°C with the primary antibodies, washed in PBS, and incubated again for 1 h at 37°C in the dark with the secondary antibodies coupled to Alexa 488 fluorophore (green) at 1/1,000. After a final wash with PBS, the slides were mounted with the DAPI‐DAKO solution. The labeling was revealed using an epifluorescence microscope (Zeiss Axio Observer Z1). The following antibodies and conditions were used: rabbit anti‐FANCD2 (ab180928 Abcam, 1/1,000); rabbit anti‐RAD51 (PC.130 Calbiochem, 1/500); and mouse anti‐α‐tubulin (T5168 Sigma 1/2,000). A minimum of 200 cells for each condition was analyzed to calculate the percentage of RAD51‐positive cells.

Detection and scoring of micronuclei were performed using an adapted cytokinesis‐block assay. Cells were incubated with cytochalasin‐B (3 μg/ml; Sigma) for 18 h before fixation. Micronuclei were scored in 40–80 bi‐nucleated cells for each condition.

Mitotic catastrophes, anaphase bridges, and post‐mitotic cells with nucleocytoplasmic bridges were scored on cells prepared for immunofluorescence analysis looking at nuclei stained with DAPI. A minimum of 10 fields (100–300 cells) were recorded for each condition to score the percentage of abnormal mitotic figures in relation to the totality of the mitoses in the microscopic fields.

### Mass spectrometry and co‐immunoprecipitation

For immunoprecipitation, HEK293 suspension cells were transiently transfected with expression plasmids of FLAG‐tagged SMC5, SMC6, and NSMCE2 using polyethyleneimine. Specifically, SMC5, SMC6, and NSMCE2 genes from hORFemone (V8.1) were transferred to mammalian expression destination plasmid pDEST26‐FLAG with LR reaction (Invitrogen). For transfection, HEK293 suspension cells were cultured in SMM 293‐TI medium (Sino Biological Inc.) supplemented with 1% Gibco FBS and 1% P/S in an 37°C incubator with shaking at 140 r.p.m.

Cells were directly lysed with NTEN buffer [20 mM Tris–HCl (pH 7.5), 150 mM NaCl, 10% glycerol, 0.5% NP40, 10 mM NaF, 1 mM PMSF, 1 μg/ml leupeptin, 1 μg/ml aprotinin] 2 days after transfection. The lysates were ultra‐centrifuged at 440,000 × *g* for 15 min, and then, the supernatant was incubated with anti‐Flag M2‐conjugated beads (Sigma‐Aldrich) for 3–4 h at 4°C. The beads were washed four times with IP buffer [20 mM Tris–HCl (pH 7.5), 150 mM NaCl, MgCl_2_ 5 mM, 10% glycerol, 0.1% NP40, 1 mM DTT, and 1 mM PMSF] and then incubated with IP buffer containing 400 μg/ml 3XFlag peptide for 1–2 h. Subsequently, the eluted complexes were analyzed by SDS–PAGE and mass spectrometry.

For immunoprecipitating SMC5‐mAID‐EGFP, 5 × 10^8^ of TK6 cells were harvested and washed by cold PBS twice. Cells were then lysed with 5 ml of CSK buffer (0.3% Triton X‐100, 100 mM NaCl, 3 mM MgCl_2_, 300 mM sucrose, 1 mM EDTA, 10 mM PIPES, 1 mM PMSF, 1× complete) for 10 min on ice. Lysates were subsequently sonicated by Bandelin SONOPULS (Sigma) (20%, 10 s, five cycles). After sonication, lysates were centrifuged at 20,000 × *g* for 5 min at 4°C. Supernatants were then incubated with 3 mg of magnetic beads protein A (Invitrogen, 10001D) which were pre‐incubated with 5** **μg of anti‐GFP (OriGene, TP401) antibody. After 2 h of incubation with rotation at 4°C, beads were washed by CSK buffer once and incubated with 1 ml of CSK buffer containing benzonase (Novagen, 70746, final: 50 U/ml) for 20 min at 37°C. Beads were then washed by CSK buffer twice and boiled with sample buffer (10% glycerol, 60 mM Tris–HCl pH 6.8, 2% SDS, 0.025% bromophenol blue) for 10 min to elute proteins.

To immunoprecipitate FANCD2, 1 × 10^8^ TK6 cells expressing SMC5‐mAID‐EGFP were lysed with 1 ml of CSK buffer. After centrifugation at 20,000 × *g* for 5 min at 4°C, supernatants were incubated with 3 mg of magnetic beads anti‐mouse IgG (Invitrogen, 11301) which were pre‐incubated with either 5 μg of anti‐FANCD2 antibody (sc20022) or mouse IgG.

Data are available upon request or uploaded as Source Data.

## Author contributions

FrR executed experiments shown in Figs 1, 2, 3 (except for Fig 3B) and EV1, EV2, EV3 and established cell lines shown in Table 2 with contributions from RK and TA. AH‐L, together with PD and FiR, designed and executed experiments shown in Figs 4A, 5, 6 and EV5. RK conducted experiments shown in Figs 3B, EV1C and 7B, C, NKJ the experiments shown in Figs 4B and EV4. XX and DX provided the results shown in Table 1 and Fig 7A. TA and MT provided useful reagents and advice. B. Szakal (acknowledged), RK, FiR, and DB made the figures starting from data provided by co‐authors. DB designed the study, supervised the work, and wrote the paper. All authors commented and provided feedback on figures and article content.

## Conflict of interest

The authors declare that they have no conflict of interest.

## Supporting information



Expanded View Figures PDF

Review Process File

Source Data for Figure 1

Source Data for Figure 2

Source Data for Figure 3

Source Data for Figure 4

Source Data for Figure 5

Source Data for Figure 6

Source Data for Figure 7
