# Supplementary material for: SMC5/6 acts jointly with Fanconi anemia factors to support DNA repair and genome stability
Source: EMBO Rep. 2019 Dec 23;21(2):e48222. doi: 10.15252/embr.201948222 (PMC7001510; doi:10.15252/embr.201948222)
Supplement: Supplementary file 1 — Expanded View Figures PDF [file EMBR-21-e48222-s001.pdf]

Expanded View Figures

Figure EV1. SMC5 function promotes normal cell cycle and prevents checkpoint-dependent G2/M delay.

A Schematic representation of the *smc5-aid-flag* modified genomic locus.
B Schematic representation of the *smc5* KO construct.
C Growth curve of WT and *smc5* cells complemented or not with chicken SMC5-HA cDNA. The experiment is carried out at the indicated temperature of 39.5°C. The data represent the means ± SD of three experiments. On the right panel, WB analysis of SMC5-HA expression after complementation. Alpha-Tubulin is used as loading control.
D Bidimensional FACS analysis is carried out to study cell cycle distribution after caffeine treatment (+Caf). Data represent means ± SD of four experiments. Asterisks indicate *P* value ≤ 0.05, as calculated by paired *t*-test.

© 2019 The Authors

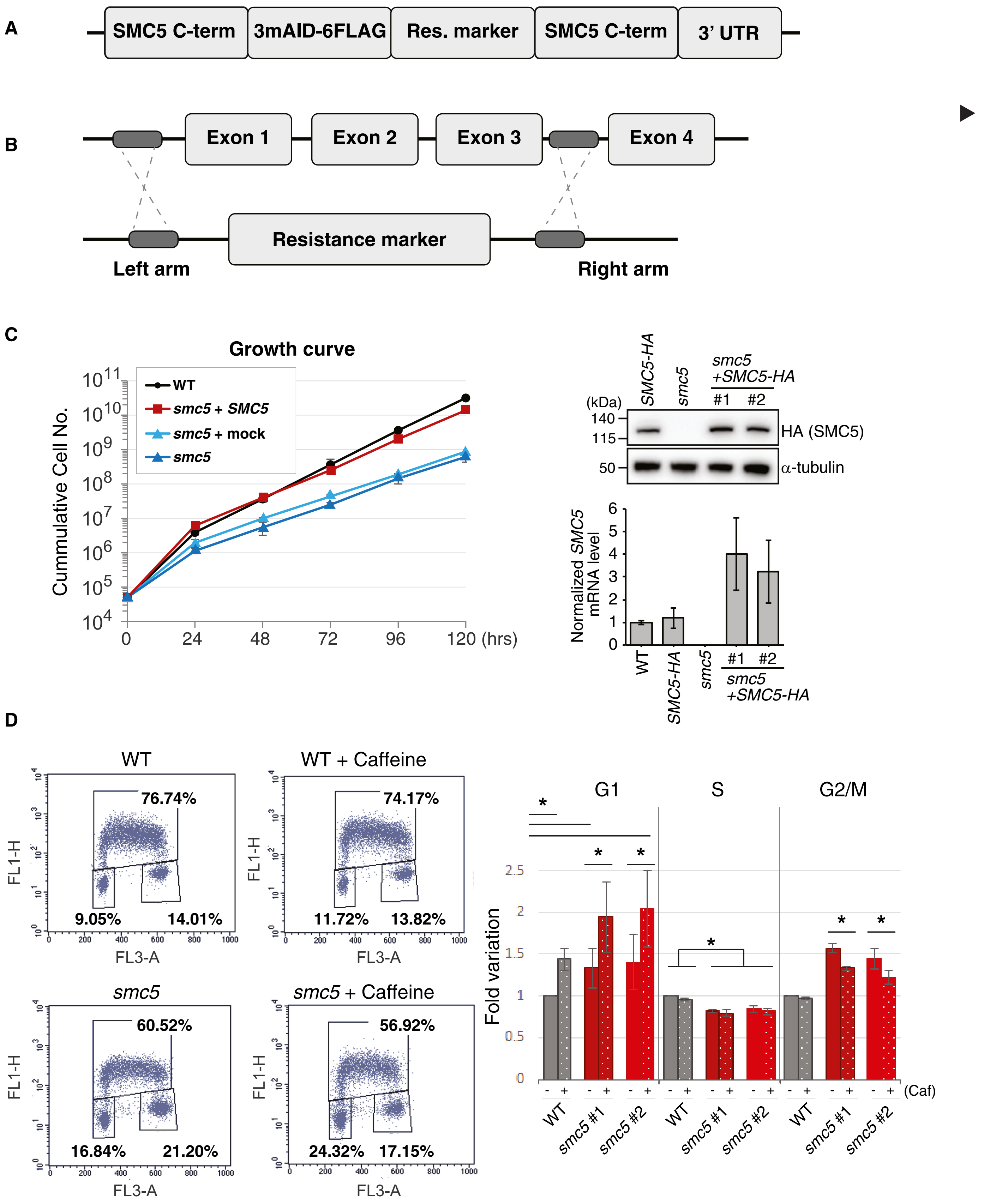
A
SMC5 C-term
3mAID-6FLAG
Res. marker
SMC5 C-term
3' UTR
B
Exon 1
Exon 2
Exon 3
Exon 4
Resistance marker
Left arm
Right arm
C
Growth curve
WT
smc5 + SMC5
smc5 + mock
smc5
Cummulative Cell No.
0
24
48
72
96
120 (hrs)
SMC5-HA
smc5
smc5 +SMC5-HA
#1
#2
(kDa)
140
115
50
HA (SMC5)
α-tubulin
Normalized SMC5 mRNA level
WT
D
WT
WT + Caffeine
smc5
smc5 + Caffeine
76.74%
9.05%
14.01%
74.17%
11.72%
13.82%
60.52%
16.84%
21.20%
56.92%
24.32%
17.15%
FL1-H
FL3-A
G1
S
G2/M
Fold variation
(Caf)
smc5 #1
smc5 #2

Figure EV1.

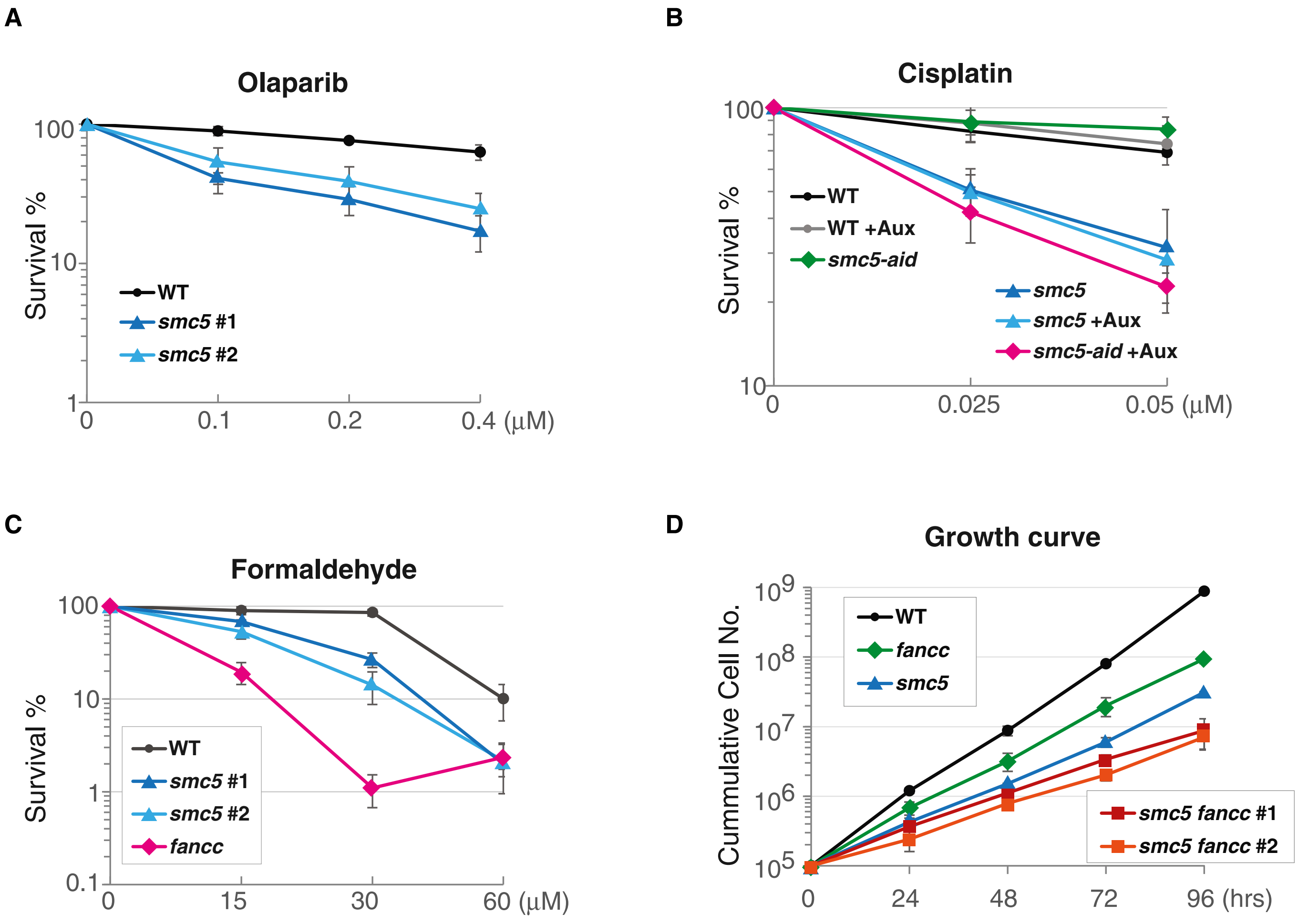

Figure EV2. SMC5 promotes tolerance to olaparib, cisplatin, and formaldehyde.

A–C Survival curve of cells of the indicated genotype toward various drugs. Data represent means ± SD of three experiments.
D Growth curve of cells of the indicated genotype. The data represent the means ± SD of three experiments.

© 2019 The Authors

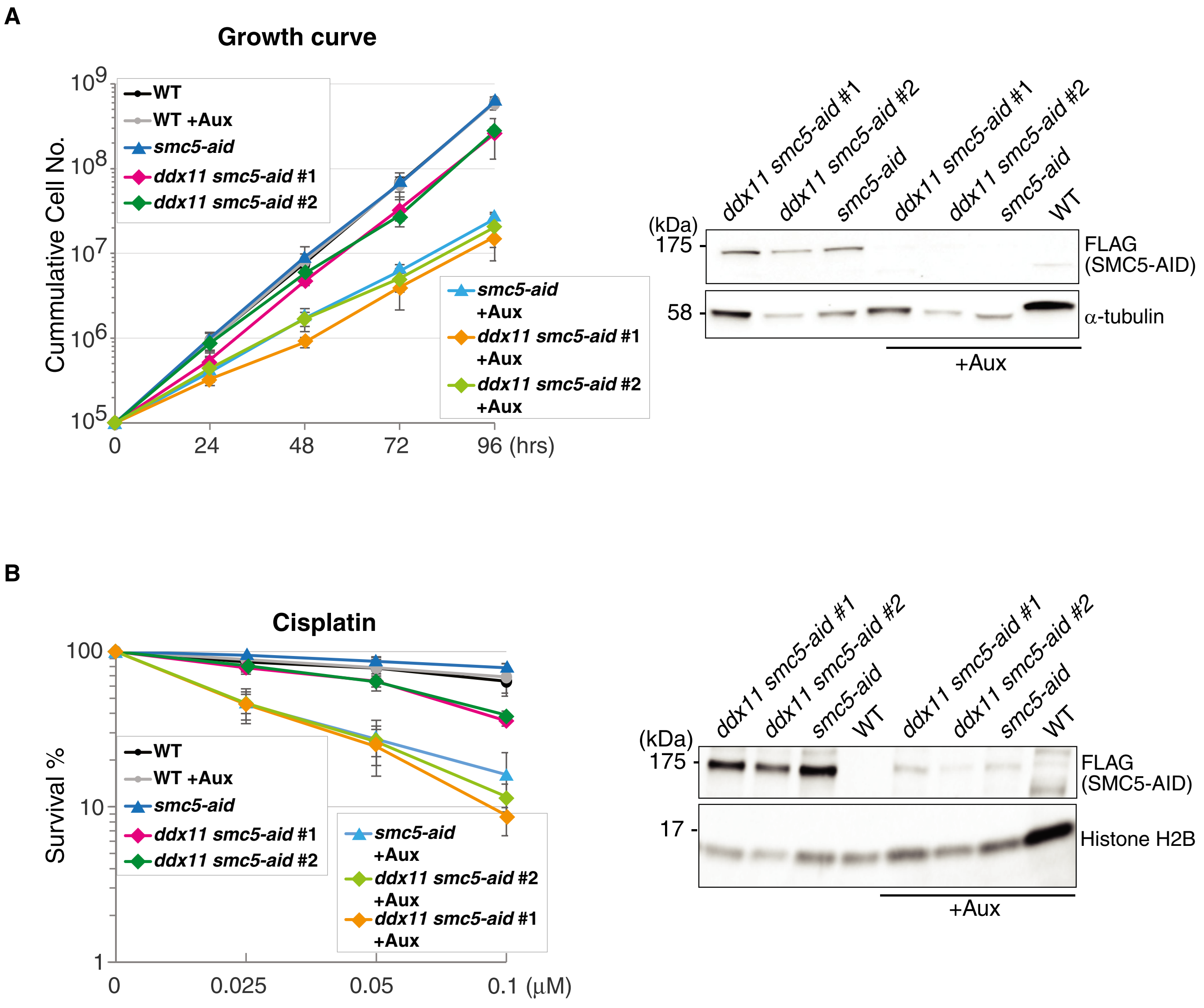

Figure EV3. SMC5 functions jointly with DDX11 to promote DNA repair.

A Left panel: Growth curve was carried out to test *ddx11 smc5-aid* cell proliferation after SMC5-AID depletion induced by auxin treatment. Data represent means ± SD of three experiments. Experiments were carried out at 39.5°C. Right panel: Western blot monitoring SMC5-AID-FLAG protein level in the presence or absence of auxin. Tubulin was used as loading control.

B Survival curve of *ddx11 smc5-aid* cells treated with different concentrations of cisplatin after SMC5 depletion upon auxin treatment. Data represent means ± SD of four experiments. Right panel: Western blot monitoring SMC5-AID-FLAG protein level in the presence or absence of auxin. Histone H2B was used as loading control.

© 2019 The Authors

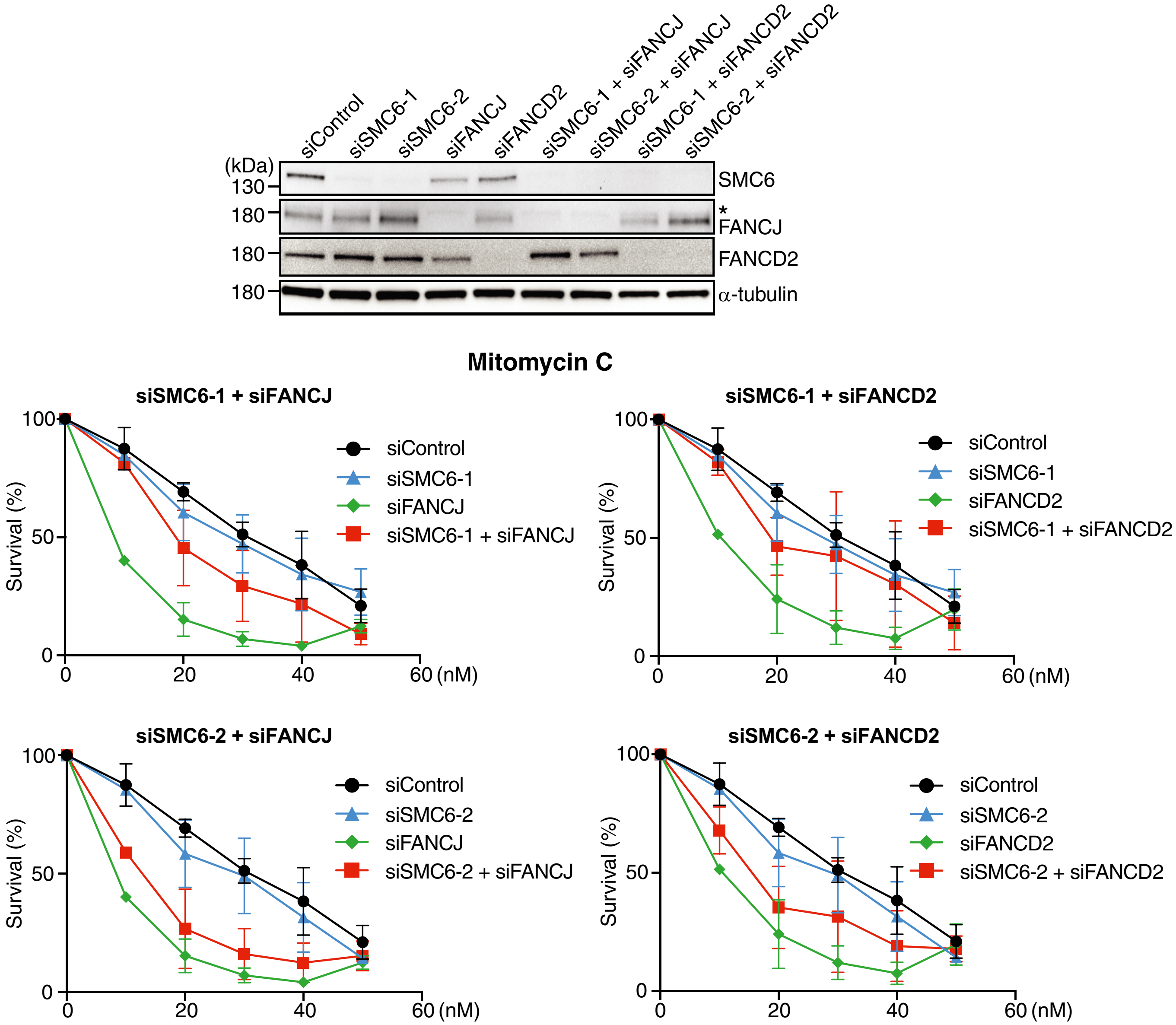

Figure EV4. SMC5/6 does not affect FANCD2 focus formation.

Left panel: representative Western blots illustrating the expression of SMC5, SMC6, and monoubiquitylated FANCD2 in protein extracts from HeLa cells transfected with the indicated siRNAs and treated 48 h after transfection with aphidicolin 0.3 μM for 12 h. Vinculin is used as loading control. Right panel: representative spreads with FANCD2 foci and DAPI staining in control and SMC5/6-depleted cells. The scale bar is 10 μm.

 © 2019 The Authors

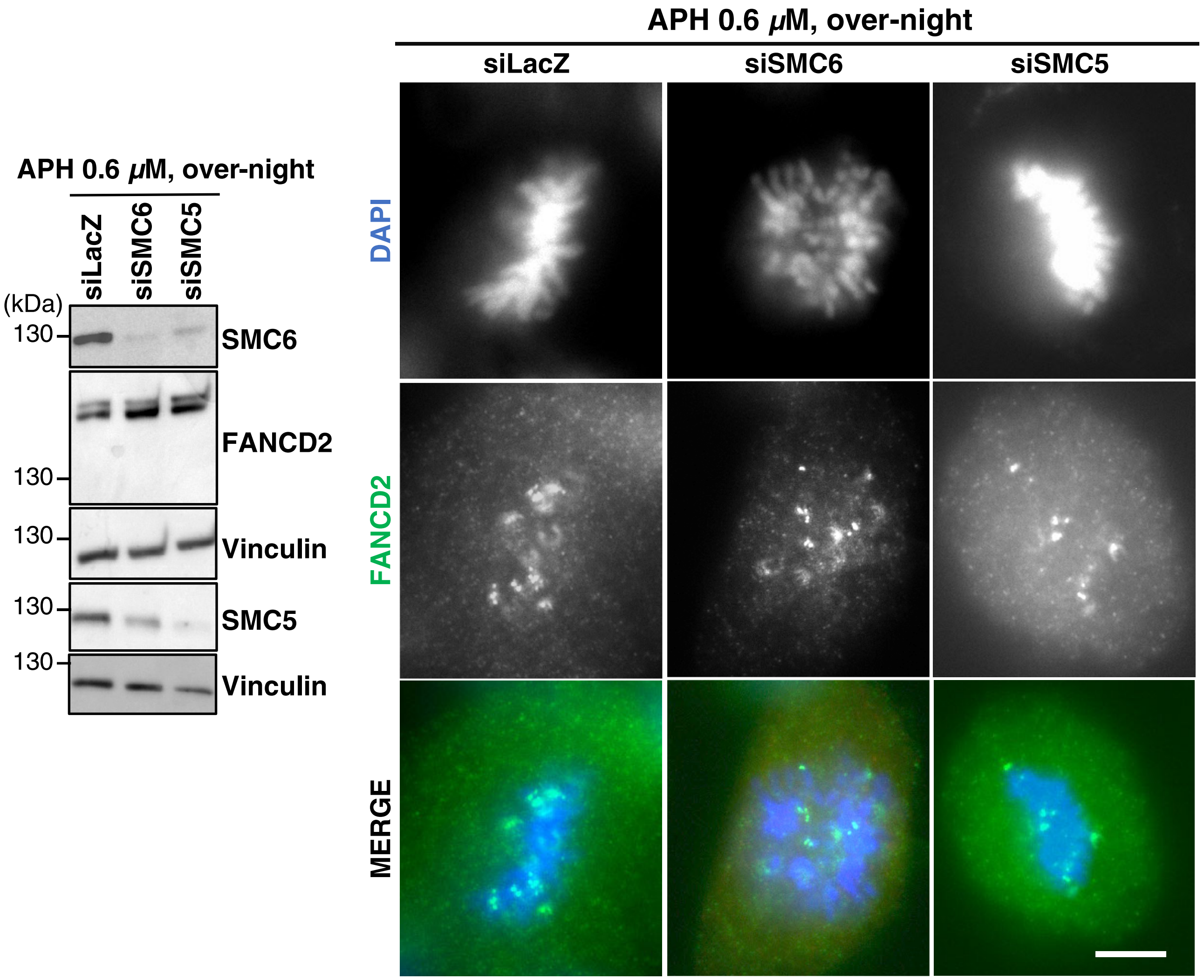

Figure EV5. SMC5/6 does not affect FANCD2 foci formation.

Left panel: representative Western blots illustrating the expression of SMC5, SMC6, and monoubiquitylated FANCD2 in protein extracts from HeLa cells transfected with the indicated siRNAs and treated 48 h after transfection with aphidicolin 0.6 μM overnight. Vinculin was used as loading control. Right panel: representative spreads with FANCD2 foci and DAPI staining in control and SMC6-depleted cells. The scale bar is 10 μm.

© 2019 The Authors
