# Supplementary figures and images for: SMC5/6 acts jointly with Fanconi anemia factors to support DNA repair and genome stability
Source: EMBO Rep. 2019 Dec 23;21(2):e48222. doi: 10.15252/embr.201948222 (PMC7001510; doi:10.15252/embr.201948222)

## Slide 1
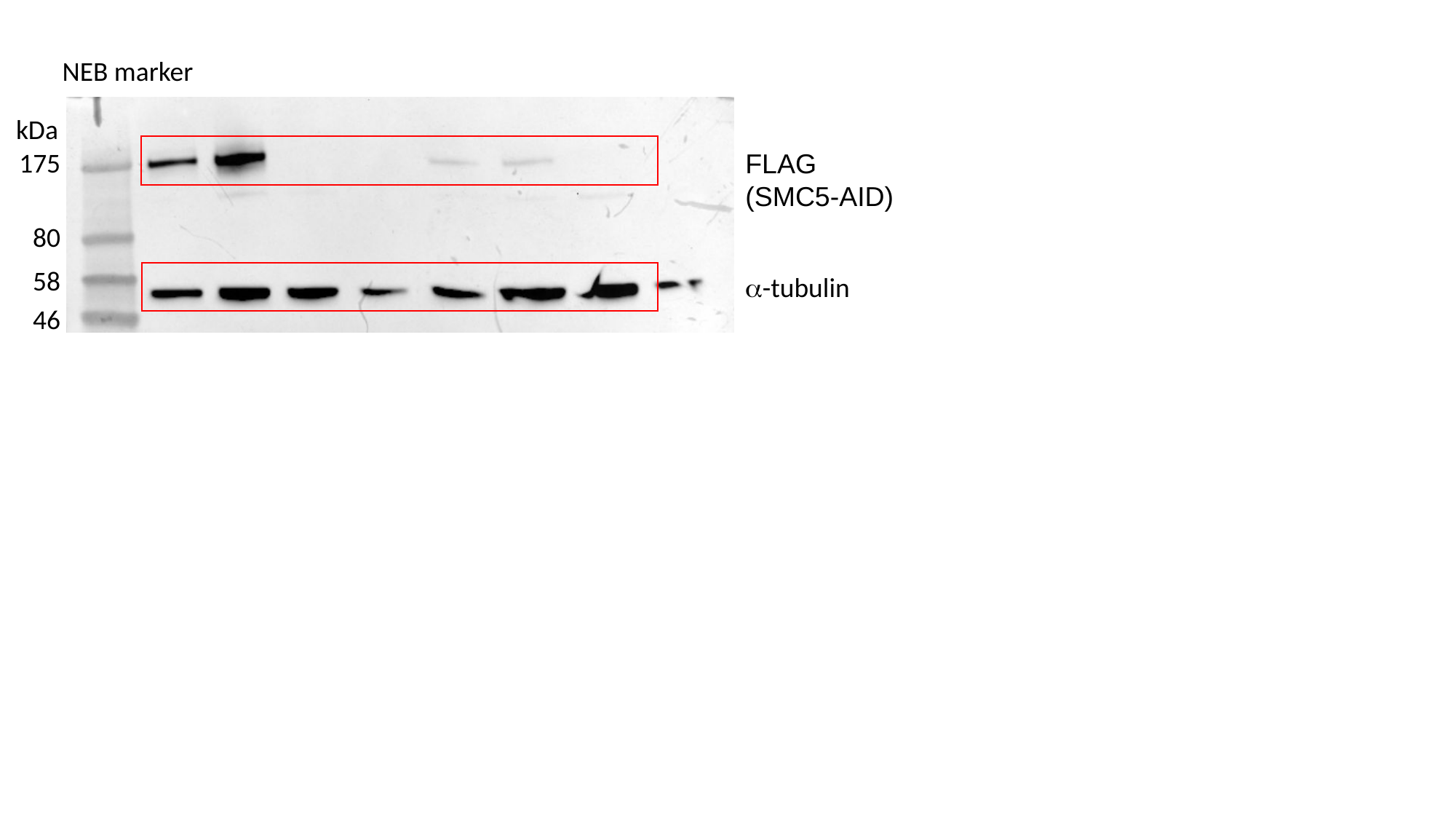

NEB marker
kDa
175
FLAG
(SMC5-AID)
80
58
a-tubulin
46

Supplement: Supplementary file 3 — Source Data for Figure 1 [file EMBR-21-e48222-s002.zip › Fig.1A.pptx]

## Slide 1
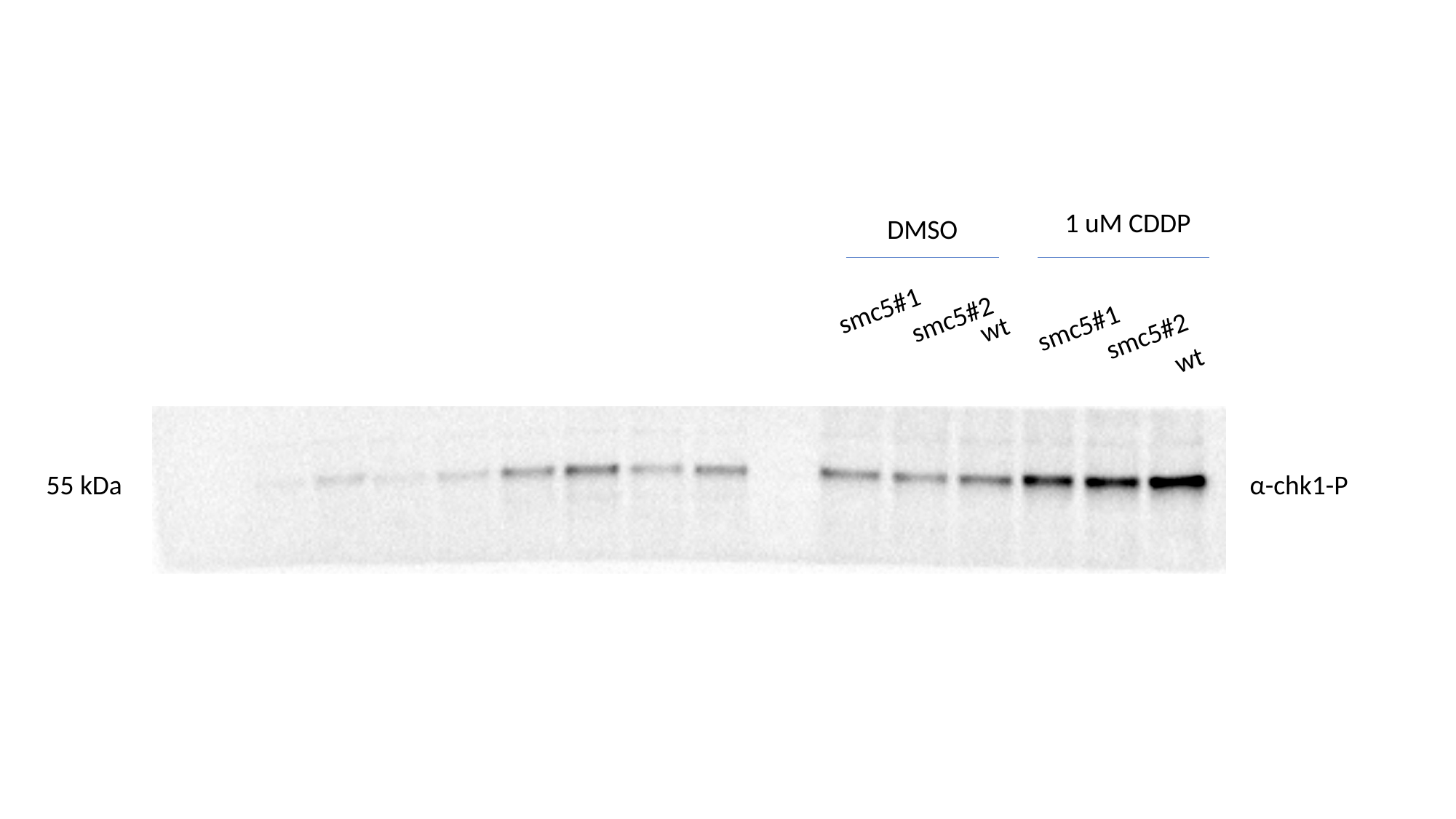

1 uM CDDP
DMSO
smc5#1
smc5#2
wt
smc5#1
smc5#2
wt
55 kDa
α-chk1-P

## Slide 2
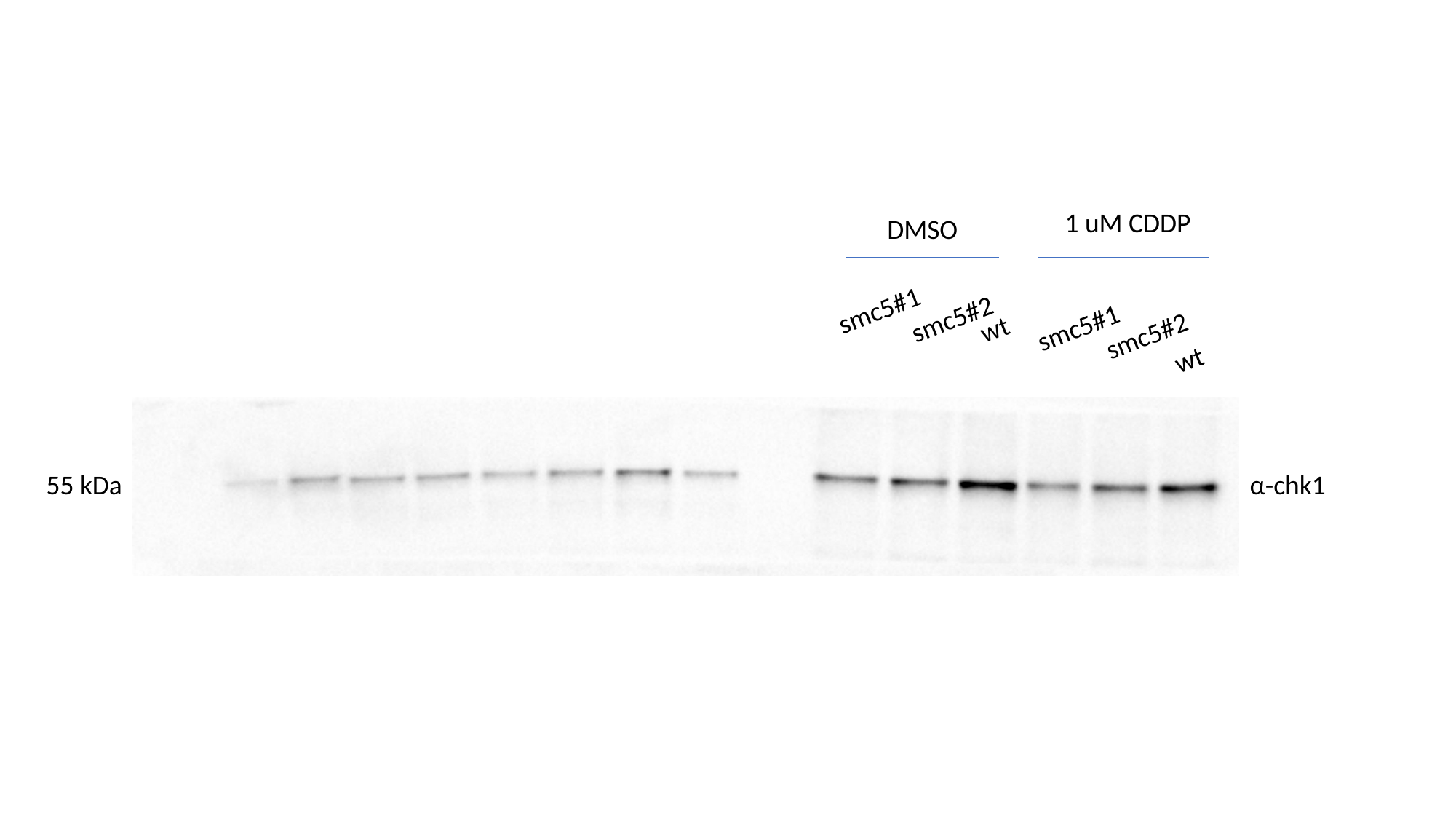

1 uM CDDP
DMSO
smc5#1
smc5#2
wt
smc5#1
smc5#2
wt
55 kDa
α-chk1

Supplement: Supplementary file 4 — Source Data for Figure 2 [file EMBR-21-e48222-s003.zip › Fig.2E.pptx]

## Slide 1
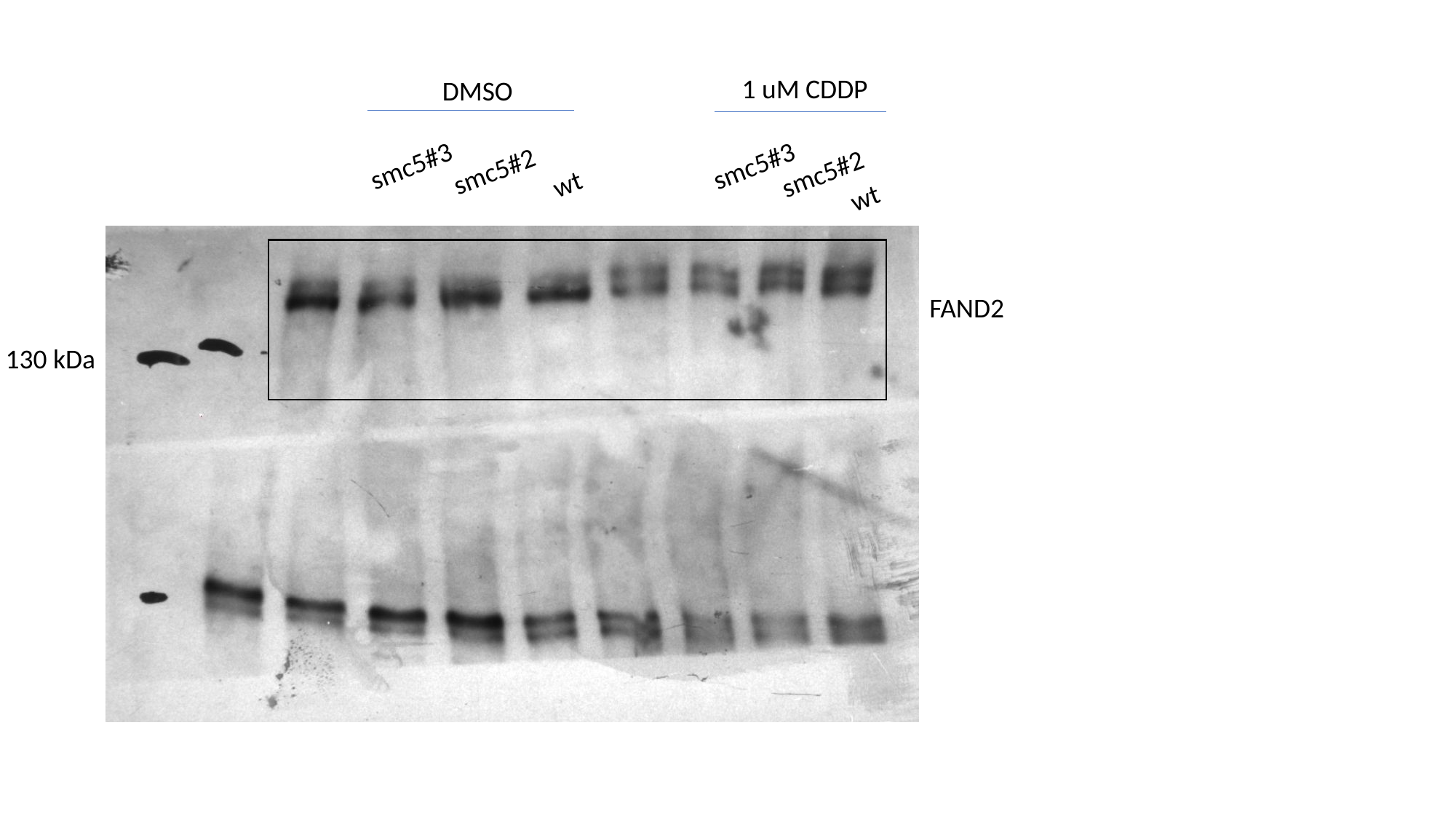

1 uM CDDP
DMSO
smc5#3
smc5#3
smc5#2
smc5#2
wt
wt
FAND2
130 kDa

Supplement: Supplementary file 4 — Source Data for Figure 2 [file EMBR-21-e48222-s003.zip › Fig.2F.pptx]

## Slide 1
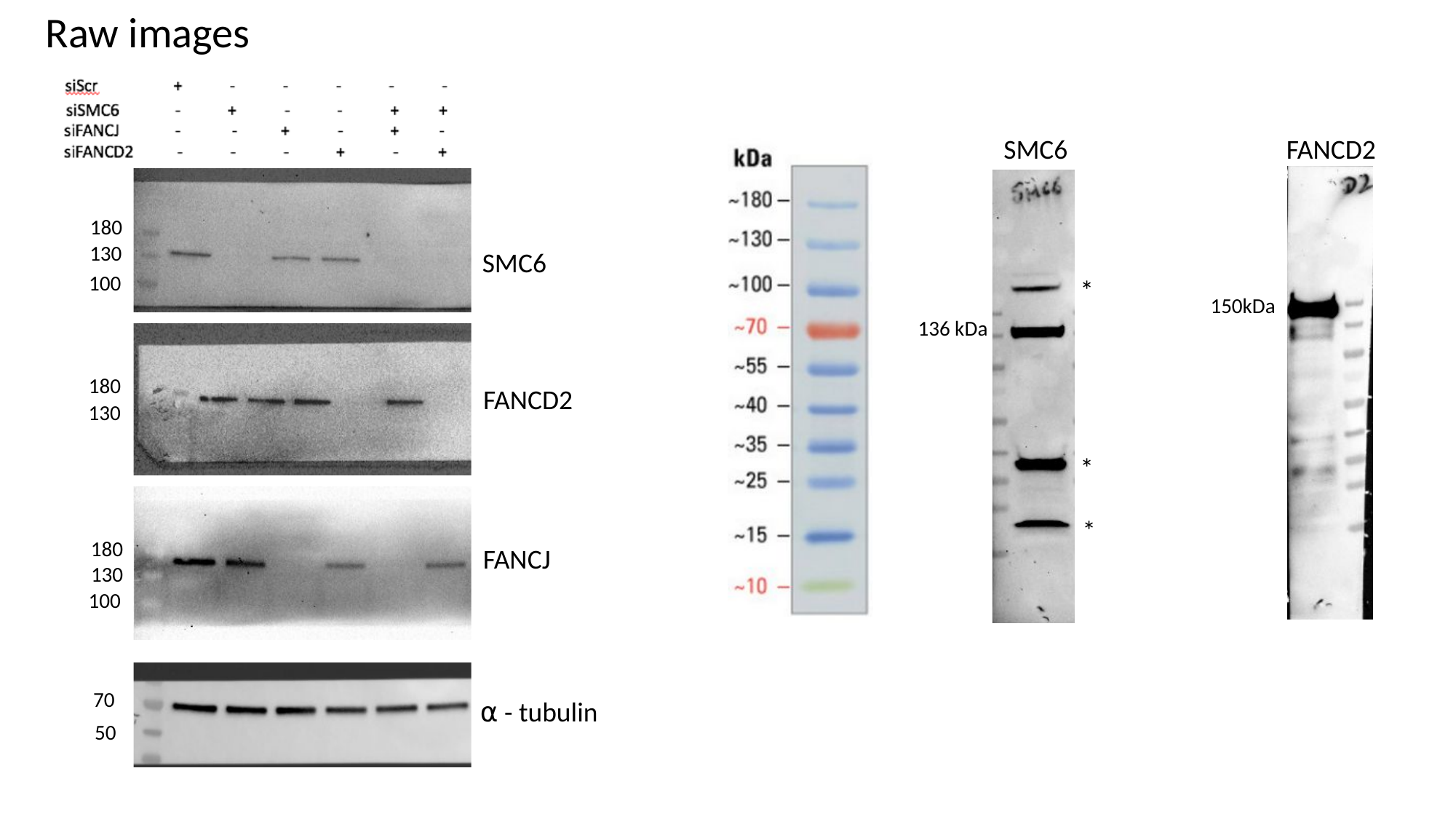

Raw images
SMC6
FANCD2
180
130
SMC6
100
*
150kDa
136 kDa
180
FANCD2
130
*
*
180
FANCJ
130
100
70
⍺ - tubulin
50

Supplement: Supplementary file 6 — Source Data for Figure 4 [file EMBR-21-e48222-s005.zip › Fig.4B_up_WB.pptx]

## Slide 1
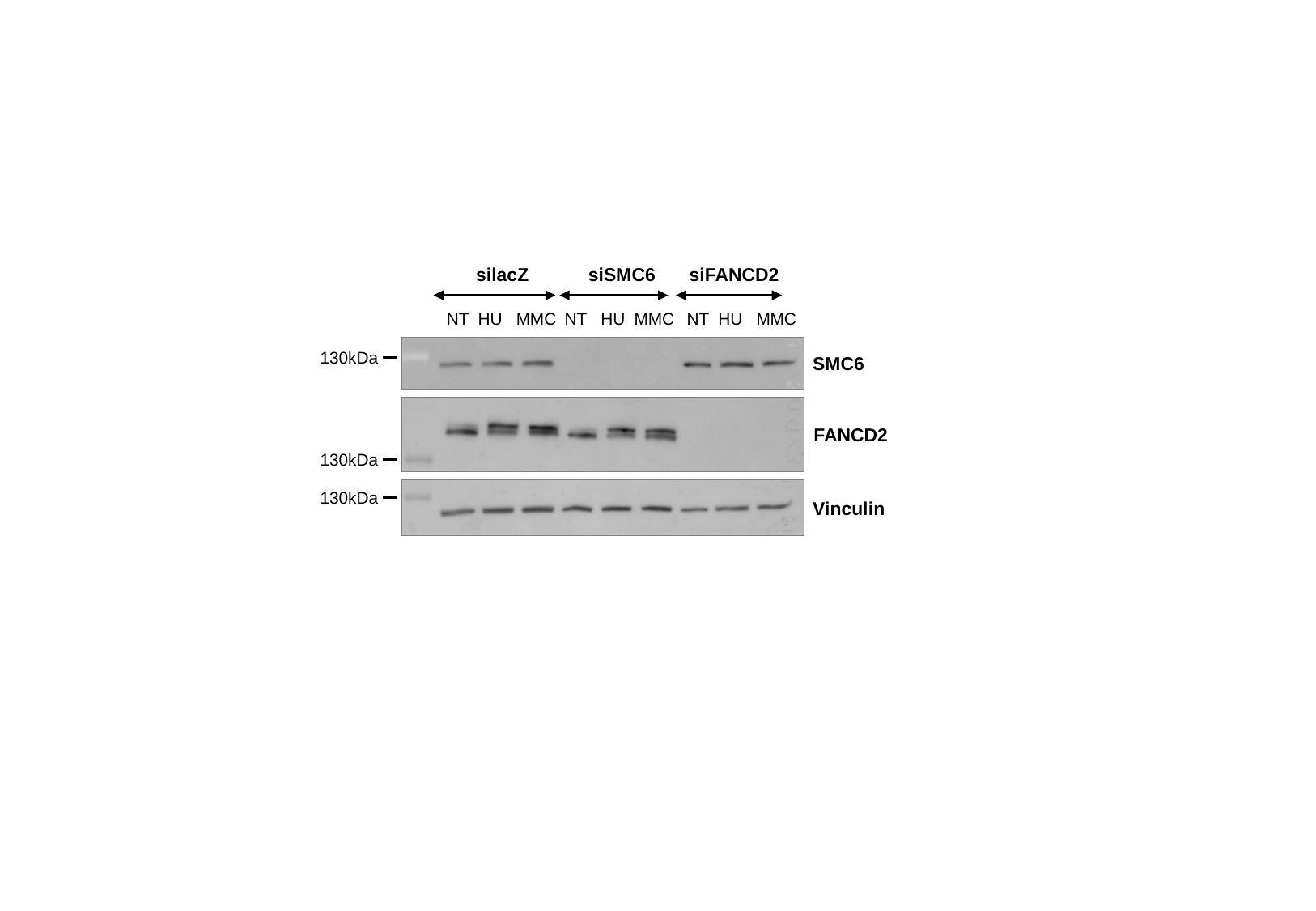

silacZ
siSMC6
siFANCD2
NT HU MMC
NT HU MMC
NT HU MMC
SMC6
FANCD2
Vinculin
130kDa
130kDa
130kDa

Supplement: Supplementary file 7 — Source Data for Figure 5 [file EMBR-21-e48222-s006.zip › Fig.5A.pptx]

## Slide 1
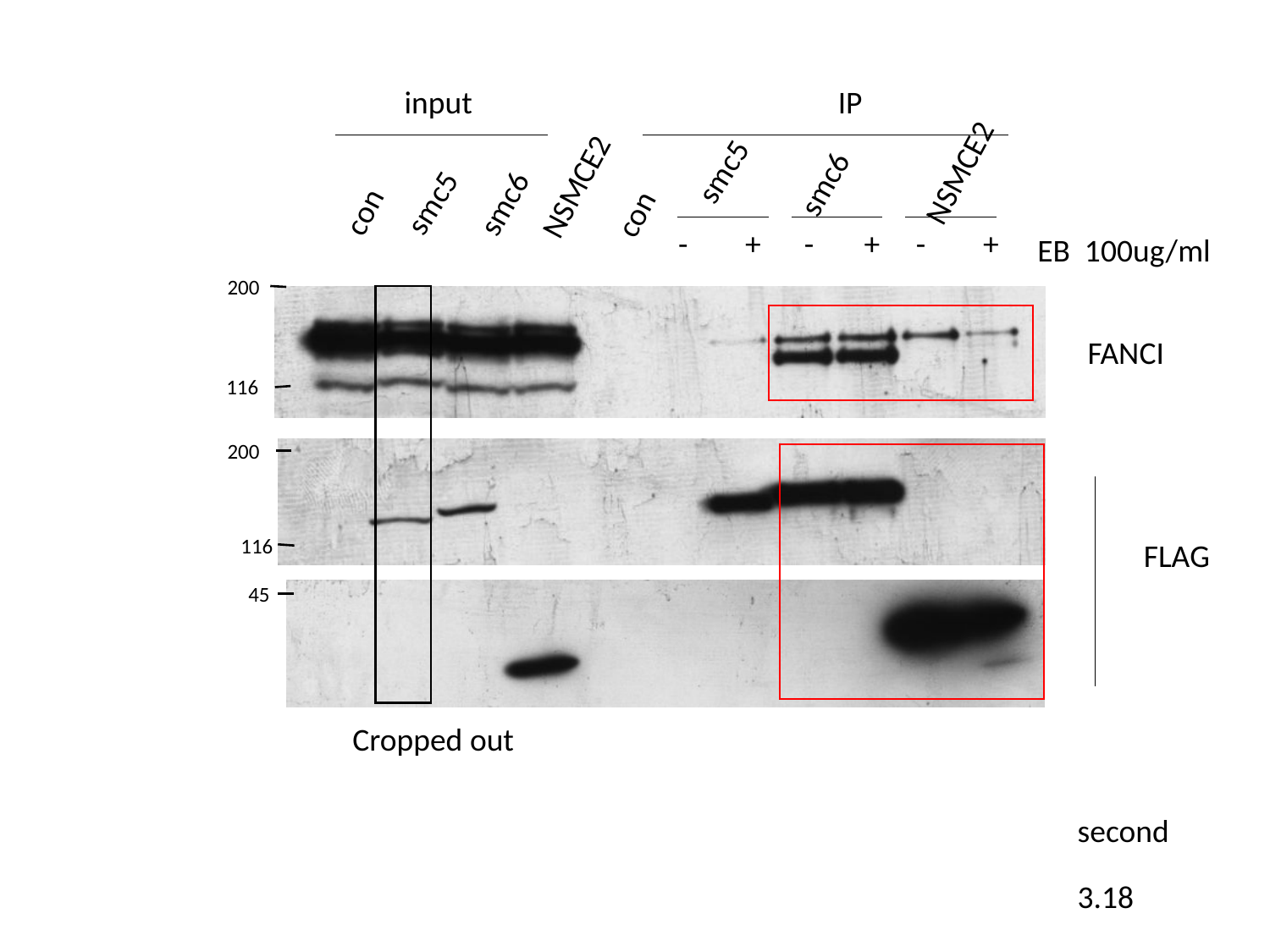

input
IP
NSMCE2
con
smc5
smc6
NSMCE2
smc5
smc6
con
- + - + - +
EB 100ug/ml
200
FANCI
116
200
116
FLAG
45
Cropped out
second
3.18

Supplement: Supplementary file 9 — Source Data for Figure 7 [file EMBR-21-e48222-s008.zip › Fig.7A.pptx]

## Slide 1
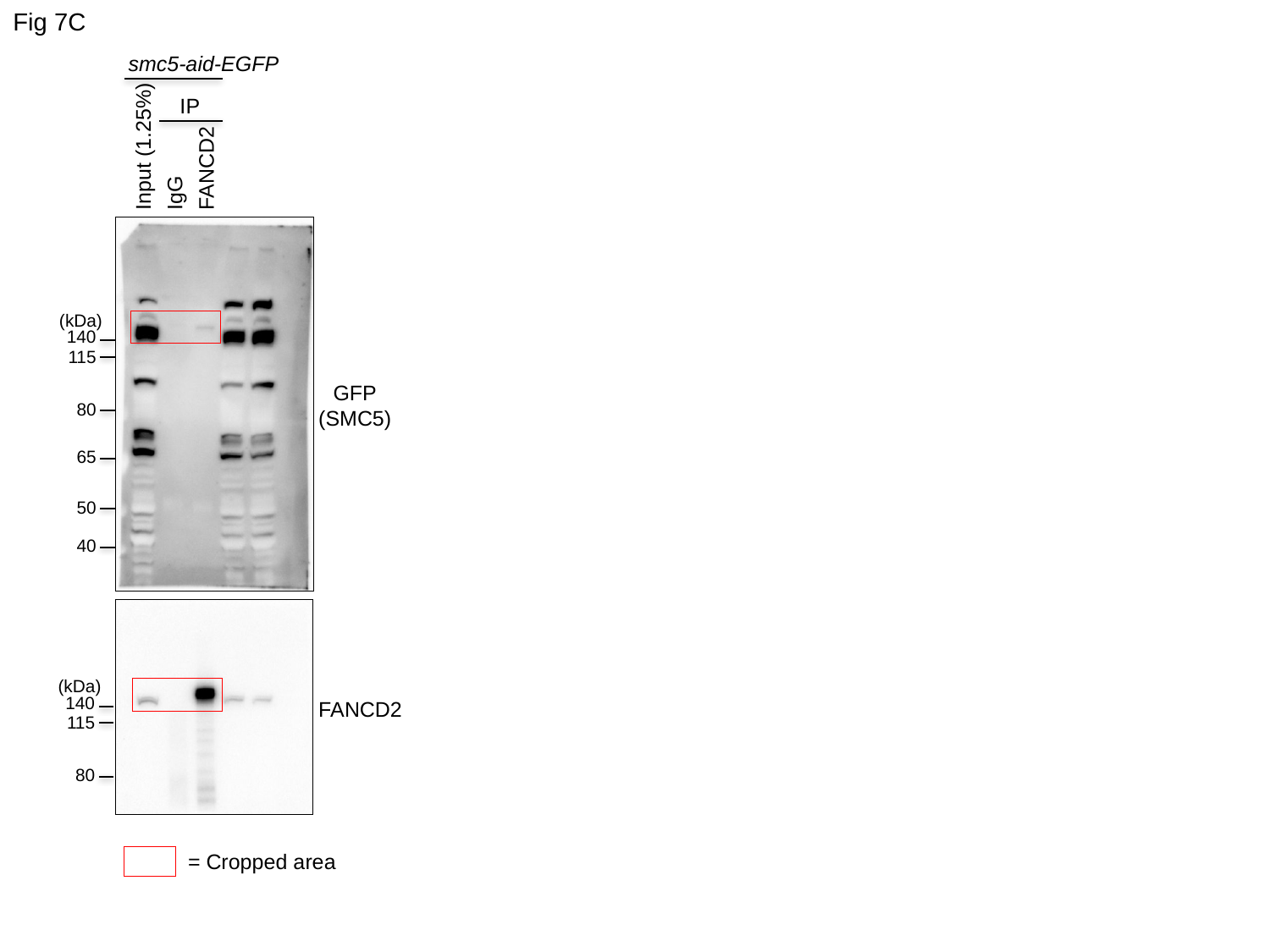

Fig 7C
smc5-aid-EGFP
IP
Input (1.25%)
FANCD2
IgG
(kDa)
140
115
GFP
(SMC5)
80
65
50
40
(kDa)
140
FANCD2
115
80
= Cropped area

Supplement: Supplementary file 9 — Source Data for Figure 7 [file EMBR-21-e48222-s008.zip › Fig.7C.pptx]
