# Supplementary material for: SMC5/6 acts jointly with Fanconi anemia factors to support DNA repair and genome stability
Source: EMBO Rep. 2019 Dec 23;21(2):e48222. doi: 10.15252/embr.201948222 (PMC7001510; doi:10.15252/embr.201948222)
Supplement: Supplementary file 6 — Source Data for Figure 4 [file EMBR-21-e48222-s005.zip › Fig.4A_left.pptx]

## Slide 1
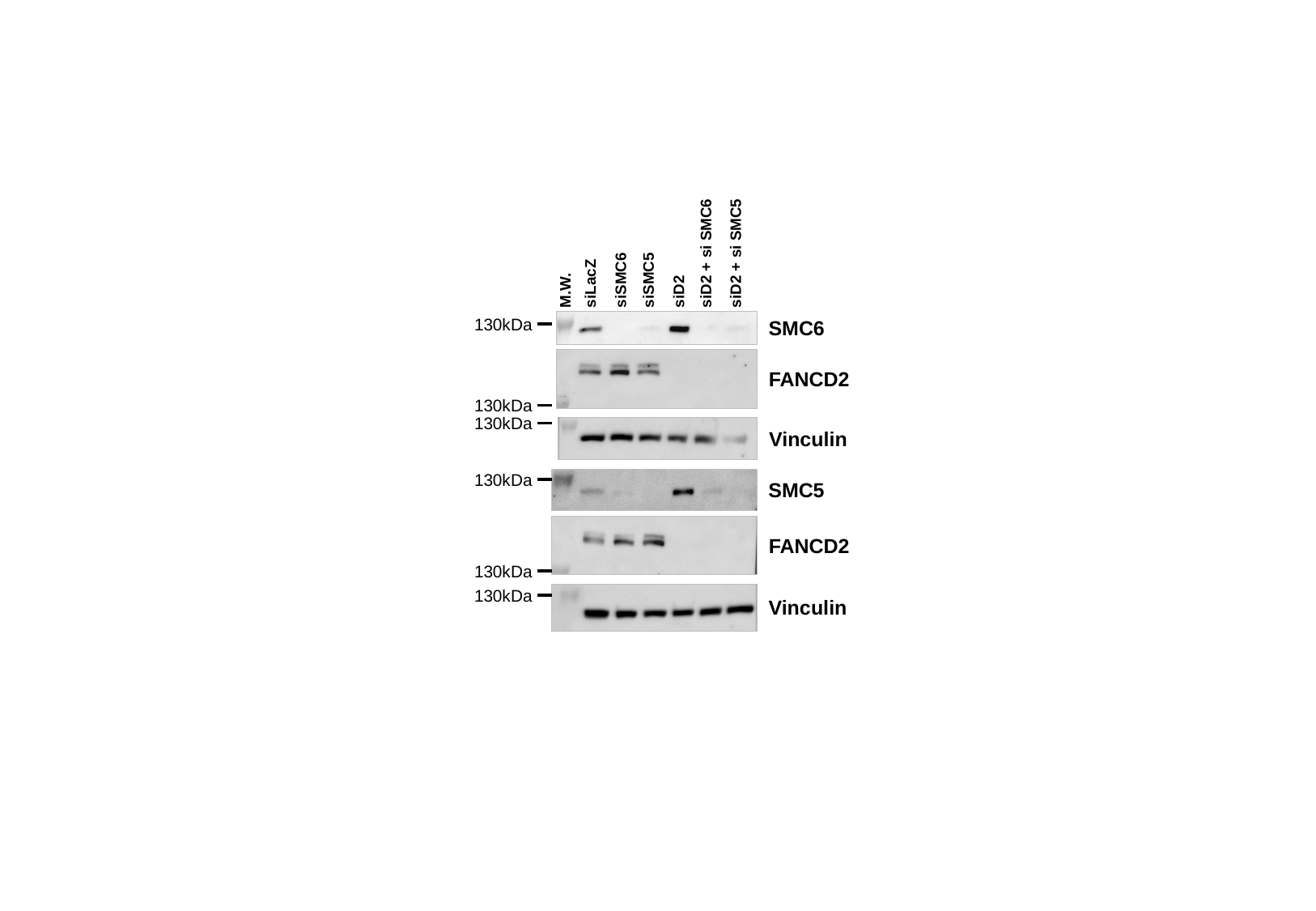

siD2 + si SMC6
siD2 + si SMC5
siSMC6
siSMC5
siD2
siLacZ
130kDa
SMC6
FANCD2
130kDa
130kDa
Vinculin
130kDa
SMC5
FANCD2
130kDa
130kDa
Vinculin
M.W.
