# Supplementary material for: SMC5/6 acts jointly with Fanconi anemia factors to support DNA repair and genome stability
Source: EMBO Rep. 2019 Dec 23;21(2):e48222. doi: 10.15252/embr.201948222 (PMC7001510; doi:10.15252/embr.201948222)
Supplement: Supplementary file 9 — Source Data for Figure 7 [file EMBR-21-e48222-s008.zip › Fig.7B.pptx]

## Slide 1
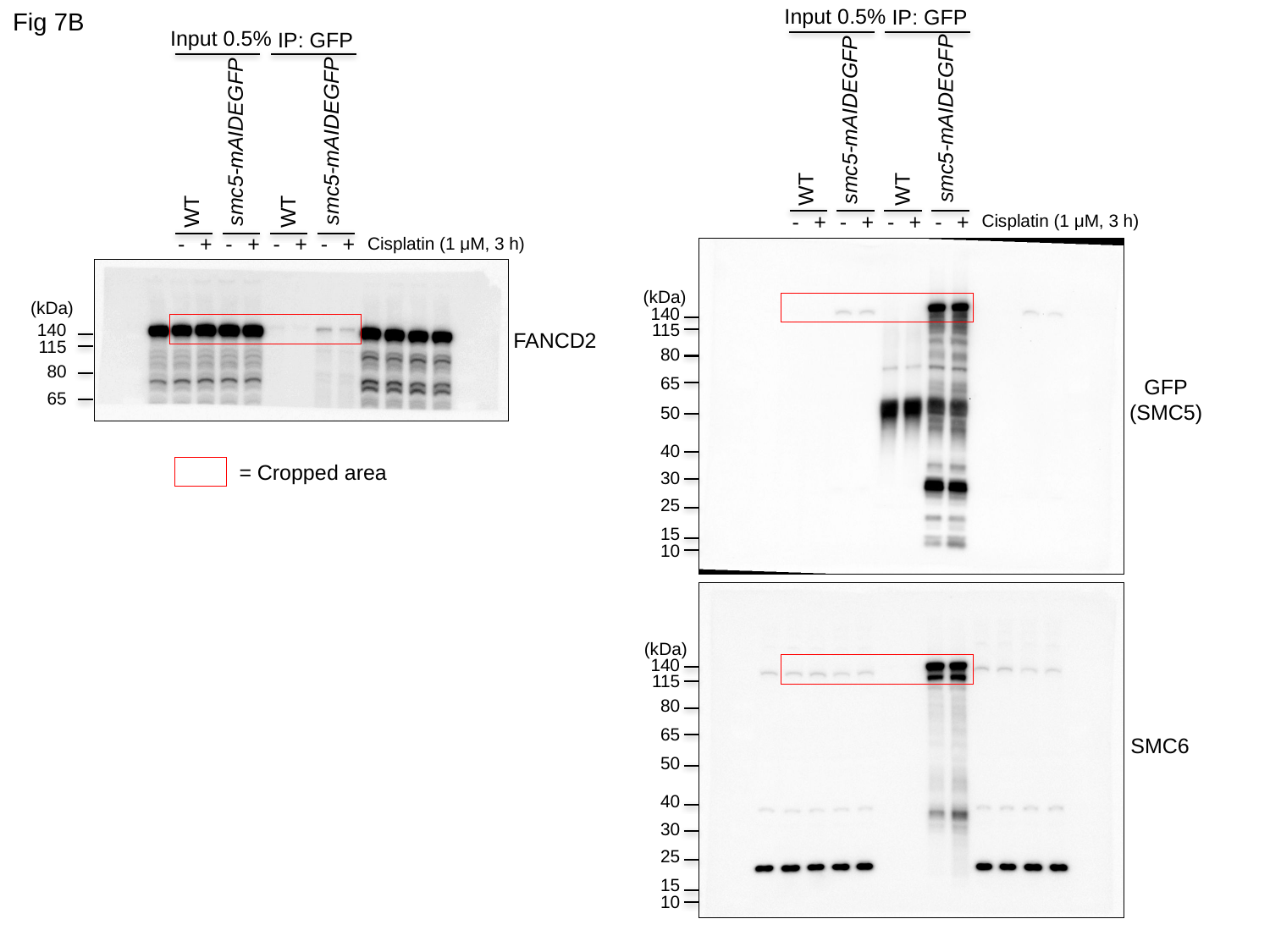

Fig 7B
Input 0.5%
IP: GFP
smc5-mAIDEGFP
smc5-mAIDEGFP
WT
WT
-
+
-
+
-
+
-
+
Cisplatin (1 μM, 3 h)
Input 0.5%
IP: GFP
smc5-mAIDEGFP
smc5-mAIDEGFP
WT
WT
-
+
-
+
-
+
-
+
Cisplatin (1 μM, 3 h)
(kDa)
140
115
80
65
GFP
(SMC5)
50
40
30
25
15
10
(kDa)
140
FANCD2
115
80
65
= Cropped area
(kDa)
140
115
80
65
SMC6
50
40
30
25
15
10
